# Cognitive and emotional regulation processes of spontaneous facial self-touch are activated in the first milliseconds of touch: Replication of previous EEG findings and further insights

**DOI:** 10.3758/s13415-022-00983-4

**Published:** 2022-02-19

**Authors:** Jente L. Spille, Stephanie M. Mueller, Sven Martin, Martin Grunwald

**Affiliations:** Paul Flechsig Institute – Centre of Neuropathology and Brain Research, Haptic Research Laboratory, University of Leipzig, 04103 Leipzig, Germany

**Keywords:** Face touch, Spectral EEG power, Attention, Working memory, Emotion

## Abstract

Spontaneously touching one’s own face (sFST) is an everyday behavior that occurs in people of all ages, worldwide. It is—as opposed to actively touching the own face—performed without directing one’s attention to the action, and it serves neither instrumental (scratching, nose picking) nor communicative purposes. These sFST have been discussed in the context of self-regulation, emotional homeostasis, working memory processes, and attention focus. Even though self-touch research dates back decades, neuroimaging studies of this spontaneous behavior are basically nonexistent. To date, there is only one electroencephalography study that analyzed spectral power changes before and after sFST in 14 participants. The present study replicates the previous study on a larger sample. Sixty participants completed a delayed memory task of complex haptic relief stimuli while distracting sounds were played. During the retention interval 44 of the participants exhibited spontaneous face touch. Spectral power analyses corroborated the results of the replicated study. Decreased power shortly before sFST and increased power right after sFST indicated an involvement of regulation of attentional, emotional, and working memory processes. Additional analyses of spectral power changes during the skin contact phase of sFST revealed that significant neurophysiological changes do not occur while skin contact is in progress but at the beginning of sFST (movement toward face and initial skin contact). The present findings clearly illustrate the complexity of sFST and that the specific trigger mechanisms and functions of this spontaneous behavior need to be further investigated in controlled, experimental studies.

**Supplementary Information:**

The online version contains supplementary material available at 10.3758/s13415-022-00983-4.

## Introduction

Spontaneously touching the own face with one or both hands (sFST) is a common everyday behavior performed by people of all ages: fetuses, infants, and young children, as well as adults (Spille et al., 2021). In the context of the COVID-19 pandemic, face touching is experiencing particular research interest, because it is associated with the transmission of pathogens to facial mucous membranes. As a result, a substantial number of studies was published that addressed the need to suppress face-touching behaviors (Chen et al., 2020; Lucas et al., 2020; Senthilkumaran et al., 2020). Spontaneously touching the own face means that the person performing the face touch pays little or no attention to the initiation and execution of the sFST, and the accuracy of remembering this behavior is poor (Hall et al., 2007; Harrigan et al., 1987). Furthermore, there is no obvious motivation underlying spontaneous face touches, and they are not intended to serve communicative or social functions as active face touches do (Spille et al., 2021).

### Trigger mechanisms and functional aspects of spontaneous facial self-touches

Previous research findings indicate that sFST occur more frequently when negative emotions, such as anxiety, tension, discomfort, or insecurity, are evoked (Carrillo-Díaz et al., 2020; D'Alessio & Zazzetta, 1986; Goldberg & Rosenthal, 1986; Harrigan, 1985; Knöfler & Imhof, 2007; Moszkowski & Stack, 2007). Recent studies have shown a positive association between trait anxiety and the number of sFST (Carrillo-Díaz et al., 2020; Carrillo-Díaz et al., 2021). In this context, emotion-regulating functions are attributed to sFST (D'Alessio & Zazzetta, 1986; Grunwald et al., 2014; Harrigan, 1985; Moszkowski & Stack, 2007; Mueller et al., 2019; Reissland, Aydin, et al., 2015a; Reissland, Francis, et al., 2015b). Furthermore, sFST may be associated with cognitive load and attentional demands (Grunwald et al., 2014; Harrigan, 1985; Mueller et al., 2019). Studies observed an increase in the frequency of sFST in tasks with increasing complexity and attentional demands (Barroso et al., 1978; Barroso & Feld, 1986). Barroso and colleagues found that higher numbers of sFST were associated with better performances in a memory task and an attentional task (Stroop Color-Word test) (Barroso et al., 1980). Assuming that performance in a task reflects attentional processes, Barroso and colleagues reasoned that sFST may be associated with increased attentional focus. In line with this, Grunwald et al. (2014) and Mueller et al. (2019) found a higher rate of sFST when distracting auditory stimuli were presented during a delayed memory task. However, Grunwald et al. (2014) reported that when distracting sounds or a working memory task were used independently from each other no increase in sFST occurred. Similarly, Densing et al. (2018) did not find an increase in sFST when inducing high stress in the arithmetic part of the Trier Social Stress Test. The various research findings demonstrate that the exact trigger mechanisms of sFST remain unknown. Moreover, most of the interpretations of sFST discussed in the literature are based solely on behavioral data obtained in observational studies. While various interpretations seem plausible, more experimental studies are required to gain substantiated insights (Spille et al., 2021).

### Neurophysiology of spontaneous facial self-touches

**Fig. 1:**
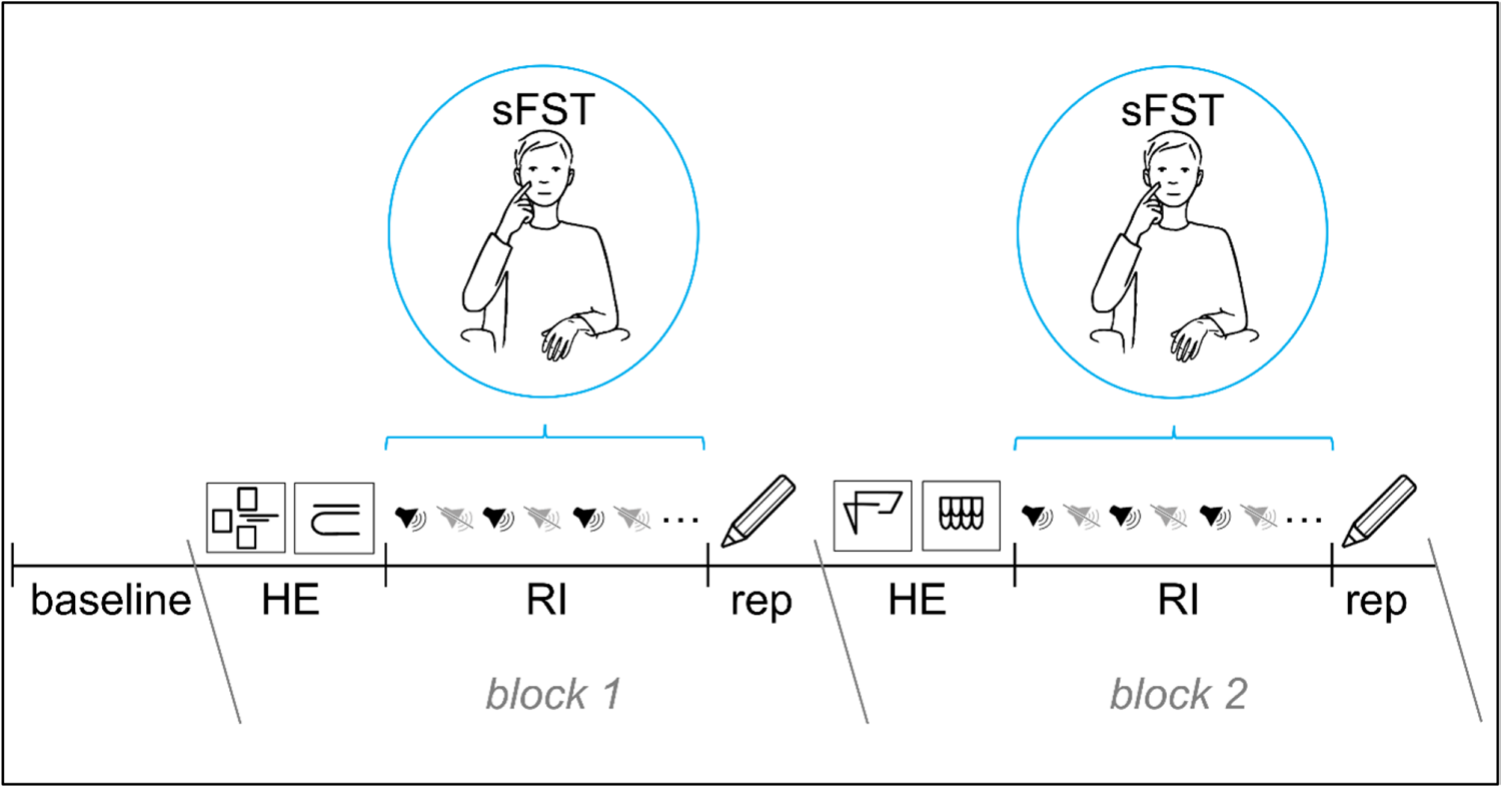
Schematic representation of the course of the experiment. After 3 minutes of rest (baseline, eyes open), two haptic reliefs had to be explored manually (HE) and subsequently remembered for a retention interval (RI) of 14 minutes. During the RI, a total of 40 distracting sounds alternated with 40 sound-free phases. Spontaneous facial self-touches (sFST) exhibited during the RI were included in the analysis. After the RI, participants were asked to reproduce (rep) the remembered stimuli on a sheet of paper. After the first block (block 1), the procedure was repeated a second time (block 2) with different relief stimuli

To date, only one study from our own research lab has investigated the neurophysiological mechanisms of sFST using electrical brain activity (electroencephalography [EEG]). In this, Grunwald et al. (2014) analyzed frequency band-specific cortical power changes before and after sFST. The authors chose an established experimental setting during which EEG changes due to working memory load have been observed before (Grunwald et al., 1999, 2001; Grunwald et al., 2004; Grunwald et al., 2014). Participants exhibited spontaneous facial self-touches during the retention interval of a delayed memory task of complex haptic stimuli when distracting sounds were played (for a schematic representation of the experimental procedure; Fig. 1). The authors observed spectral power changes in the theta band indicating that sFST serve brain regulatory functions and do not merely represent displacement activities (Grunwald et al., 2014). After exploration of haptic stimuli, theta power increased compared with baseline, which has been interpreted as a consequence of the increased memory load due to the storage of the haptic information (Grunwald et al., 2014). Shortly before sFST occurred, theta power decreased. According to the authors, this finding indicates that the distracting sounds during the retention interval were interfering with the maintenance of the memory load. In turn, internal contemplation about the fading memory may have led to emotional reactions. After sFST, theta power returned to the same level as where it had been after haptic exploration. According to Grunwald and colleagues, the increase in the theta band shortly after sFST may represent, on the one hand, successful refocusing of attention and thus maintenance of working memory content. The authors have suggested that the increase in spectral power in the theta band may reflect emotion regulation processes in response to distracting and negative external stimuli. In addition to spectral power changes in the theta band, the authors found significant changes in the beta and gamma bands. Analysis of the beta band showed significant power increases both after haptic exploration and sFST, which the authors have attributed to post-movement beta synchronization (Grunwald et al., 2014). The spectral power of gamma frequency showed, in parallel with the changes of spectral theta power, significant increases after haptic exploration as well as significant decreases before sFST. The increased activity of spectral gamma power has been discussed as a phase coupling process between theta and gamma band oscillations in the context of memory tasks. The spectral power of the alpha frequency did not show any significant changes over the course of the experiment (Grunwald et al., 2014). In addition, the authors analyzed spectral power changes before and after instructed facial self-touches, which had to be performed at request of the investigator in a reference situation without additional working memory demands. No significant changes were detected in any of the frequencies when the spectral power before and after instructed facial self-touches were compared (Grunwald et al., 2014).

### Present study

Although sFST is a common behavior and has become an increasing focus of research due to the risk of infection transmission, no other neurophysiological studies on the phenomenon of sFST are available to date. Therefore, and in order to test the theoretical assumptions regarding regulatory functions of sFST, the present study was designed to replicate the results from Grunwald et al. (2014). In a larger sample, we want to examine whether sFST are associated with specific changes in electrical brain activities that indicate the presumed involvement of sFST in the regulation of attentional, emotional, and working memory processes. Because there is a paucity of brain physiological data on sFST to date, a close replication with extension design was used (Brandt et al., 2014). The methods of the study by Grunwald et al. (2014) were reproduced as accurately as possible. However, to extend the generalizability of the results and to remedy methodological deficiencies from the previous study, extensions were made with regard to methodological approaches as well as analytical procedures. Thus, the use of triaxial accelerometers allowed more precise recordings of the temporal structure of sFST and thus an analysis of EEG activity during the skin contact phase of sFST. In addition, the study by Grunwald et al. (2014) did not analyze slow wave activities of the delta frequency. However, since delta band activity has been discussed in relation to cognitive as well as emotional processes (Güntekin & Başar, 2016; Knyazev, 2012), spectral changes in the delta band were considered in the present study. This also is important because neurophysiological sFST research is in its early stages of development, and therefore, an explorative approach is important in order not to miss important findings that would contribute to the further development of the theory on sFST.

### Cortical activity before and after sFST during delayed memory tasks (Hypotheses 1a-c, replication of previous study results)

**Fig. 2:**
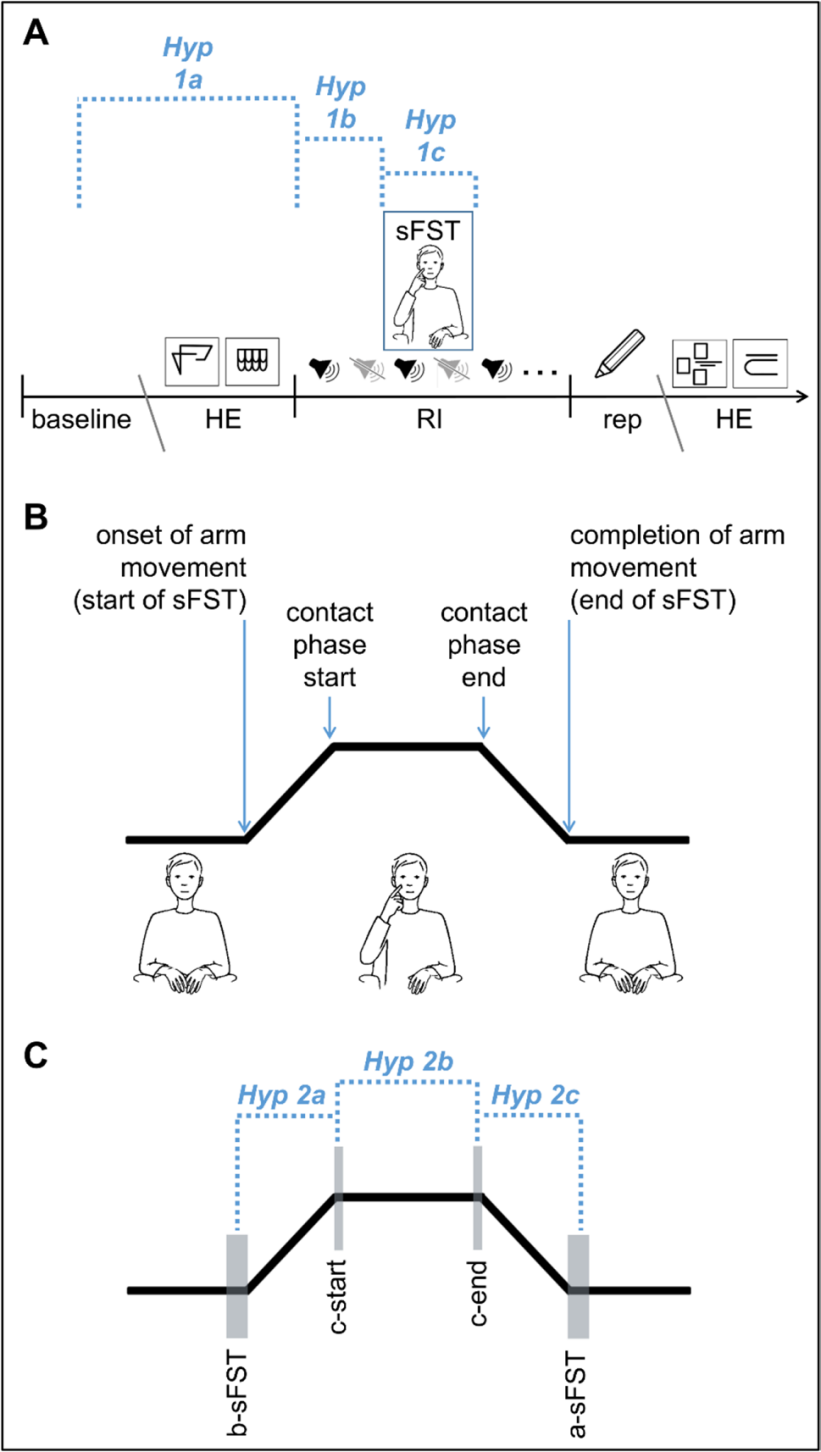
**a** Schematic representation of the spectral EEG power comparisons (dotted lines) between the 3-minute baseline and 3 s after haptic exploration (HE) (Hyp 1a), between 3 s after HE and 3 s before spontaneous facial self-touches (sFST) (Hyp 1b), and between 3 s before and 3 s after sFST (Hyp 1c) (Adapted from Grunwald et al., 2014, Figure drawn by C. Maiwald). **b** Schematic representation of the course of a sFST with markings of the start of sFST (onset of arm movement), skin contact phase (start and end), and end of sFST (completion of arm movement). **C.** Schematic representation of the spectral EEG power comparisons (dotted lines) between 3 s before spontaneous facial self-touch (b-sFST) and the first 500 ms of skin contact (c-start) (Hyp 2a), between the first 500 ms (c-start) and the last 500 ms (c-end) of skin contact during a sFST (Hyp 2b), and between the last 500 ms of skin contact (c-end) and 3 s after sFST (Hyp 2c)

To replicate the findings of Grunwald et al. (2014), frequency band-specific power differences in the delta, theta, alpha, beta, and gamma bands were calculated by comparing the mean spectral absolute powers between consecutive events of the experiment (Fig. 2).

In hypothesis 1a, we expected to find spectral power increases when comparing the resting period (baseline) and a 3-s period after haptic exploration (aHE). In line with the results of Grunwald et al. (2014), we expected significant increases in the spectral power of theta, beta, and gamma during 3-s aHE relative to the baseline due to increasing memory load. Investigations of the delta frequency band have shown increases in spectral delta power after the presentation of memory sets during working memory tasks (Fernandez et al., 2002; Harmony et al., 1996; Harmony et al., 2004). Experimental data also have indicated a similarity in the functional correlates of delta and theta oscillations in relation to cognitive processes (Başar et al., 2001). We therefore expect spectral delta power to also increase aHE.

In hypothesis 1b, we expect to find spectral power decreases when comparing a 3-s period aHE and a 3-s period before sFST. The results of Grunwald et al. (2014) showed a decrease in theta and gamma power shortly before sFST occurred. For comparison of 3-s aHE and a period of 3-s before sFST, we therefore expected to find a decrease in theta and gamma power. For the delta frequency band, similar to the changes in the theta band, we assumed that a distraction of attention during the delayed working memory task is accompanied by a decrease in spectral power before sFST.

In hypothesis 1c, we expected to find spectral power increases in a 3-s period after sFST compared with a 3-s period before sFST. Performance of sFST was thought to be associated with a refocusing of attention on working memory process. Grunwald et al. (2014) observed that the theta and beta band showed similar increased spectral power values after sFST as aHE. In line with these results, we expected theta and beta to increase after sFST. Research findings on delta power have suggested that there is a link between increased delta activity and cognitive processes related to attention (Harmony, 2013; Knyazev, 2012) as well as emotional processes (Güntekin & Başar, 2016; Knyazev et al., 2009). Therefore, an increase in spectral power after sFST is expected for the delta band as well.

### Neurophysiological changes during the skin contact phase of spontaneous facial self-touches (Hypotheses 2a-c, extension of previous findings)

Grunwald et al. (2014) chose artifact-free segments of 3 s before the sFST started and 3 s after the sFST ended to investigate spectral changes in the context of sFST. Based on these analyses, we know that neurophysiological changes occur between 3 s before sFST and 3 s after sFST. However, for a better understanding of basic mechanisms of sFST, it is necessary to investigate brain physiological processes that take place during sFST. In the present study, in addition to EMG sensors, triaxial accelerometers were implemented, enabling a precise offline analysis of the motion sequence of sFST. By recording the EMG and EEG signals in parallel, it was possible to distinguish the different phases of a facial self-touch (for a schematic representation of an EEG segmentation for sFST see Supplementary Fig. S1).

To extend the original study, we wanted to investigate the specific changes in the EEG that occur during the skin contact phase of sFST. By definition, sFST is the touching of one's own face with one's own hand or fingers, which is why we focus on the skin contact phase in our analysis. To our knowledge, no studies have investigated neurophysiological parameters during skin contact of either spontaneous or active self-touches before. Therefore, we captured the dynamic changes in spectral EEG parameters before, during, and after skin contact of sFST. In hypothesis 2a, we expected to find spectral power changes when comparing a 3-s period before sFST and the first 500 ms of the skin contact phase, in which the facial skin was initially stimulated by touch. In hypothesis 2b, we expected to find spectral power changes when comparing the first and the last 500 ms of the skin contact phase. In hypothesis 2c, we expected to find spectral power changes when comparing the last 500 ms of the skin contact phase and the 3-s period after sFST. For a schematic representation of the hypotheses 2a-c, see Fig. 2.

## Materials and Methods

### Participants

Sixty healthy volunteers took part in the experiment (30 females; age: mean [M] = 25.72 years, standard deviation [SD] = 3.05; age range 20–35 years). All test subjects were right-handed according to a test of handedness (Oldfield, 1971). None of the participants were taking medications that affect the central nervous system. All participants were naive to any kind of neurophysiological and EEG examinations. This was necessary to ensure that participants behaved naturally during the retention interval (RI). Test subjects with EEG experience would usually have learned not to move during EEG measurements. Fifty-four of the 60 participants performed at least one sFST at some point in the experiment. A subgroup of 45 participants performed sFST during the RI. The EEG data of one participant had to be excluded due to strong artifacts. Thus, the EEG-data analyses were performed based on whole data sets of 44 participants (25 males/19 females) who performed sFST during the RI. Participants were told that they would participate in an experiment concerning memory effects of haptic exploration. After participants finished the experiment, the goal of the study was unmasked. The study was approved by the Ethics Committee of University of Leipzig Medical Faculty. All participants gave written, informed consent. Participants were paid for participation (10€/h).

### Experimental design

The data for the present study were gathered in an experiment investigating sFST during a delayed memory task of complex haptic stimuli (sunken reliefs) (Grunwald et al., 1999, 2001, 2004, 2014). The same experimental setting has been successfully used by Grunwald et al. (2014) to induce sFST. In the present study, the same material and experimental conditions were used as in the aforementioned study. In order to avoid methodological deficiencies from that study, extensions were made with regard to methodological approaches as well as analytical procedures.

The neurobiological analysis of spontaneous, unpredictable, natural behaviors is inherently limited with respect to the trials that can be obtained in laboratory studies. In order to increase the number of interpretable trials in spontaneously occurring behavior, there are few possibilities, including, for example, increasing the duration of the study, tightening the experimental conditions (i.e., a reinforcement of distressing factors and/or memory load) or increasing the number of participants. Because the first two possibilities are difficult to realize for ethical reasons, we chose to increase the number of participants in the present study. Compared with the study of Grunwald et al. (2014), in which 14 participants were tested and 71 sFST trials were included in the analysis, the number of participants in the present study was increased to 60 to obtain a higher total number of sFST trials to be analyzed.

The experiment consisted of two experimental blocks. In each of the two experimental blocks, the participants had to explore two haptic stimuli (HE), remember them for a RI of 14 minutes, and subsequently draw them on a piece of paper (rep). Distracting sounds (e.g., baby crying, explosion, siren) from a free database as well as from the database of International Affective Digitized Sounds (IADS-2) (Bradley & Lang, 2007) were presented during the RI. A detailed description of the sounds is given in the supplemental material. Between the single sounds, there were sound-free phases. Within each RI, 40 sounds and 40 sound-free phases alternated with each other. Across participants, a total of 60 different sounds were played randomly. The durations of the sounds and sound-free phases varied between 7 and 13 s to prevent habituation and anticipation effects. After the first experimental block (HE of 2 stimuli – RI of 14 minutes – rep of 2 stimuli) the procedure was repeated a second time with two different haptic stimuli (Fig. 1).

**Fig. 3:**
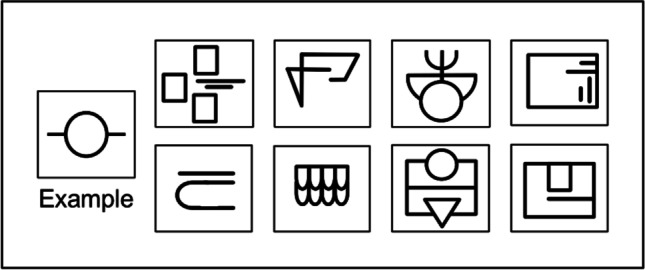
Sunken relief stimuli and example stimulus. The participant practiced manual exploration on the example stimulus before the experiment began. Each participant was randomly assigned four (two in one block) of the above pictured relief stimuli to be explored during the experiment (Graphic from Mueller et al., 2019, CC BY 4.0)

Participants were seated in a comfortable armchair with the holding equipment (for the haptic relief stimuli) in front of them. Before the experiment began, the procedure was

explained to the participants and one example stimulus as well as three example sounds were presented. Grunwald et al. (2014) used eight example sounds. The sounds were only presented to prepare the participants for the upcoming experiment and to adjust the volume of the loudspeakers. Therefore, the number of example sounds was reduced not to cause any effect by the distracting sounds before the actual experiment began. When the participant had no more questions, the experiment began with a resting phase. Grunwald et al. (2014) applied a baseline of 10 minutes. To avoid unnecessarily lengthening the experimental procedure, the baseline duration was shortened to 3 minutes in the present study. The participants sat quietly and fixated a black dot with their eyes. After rest, the experiment proper started with the haptic exploration task. An opaque screen obscured the participant’s hands and the stimulus from vision during exploration. Participants were allowed to explore the reliefs as long as they pleased, with one or both hands. Each sunken relief was milled into a plastic plate of 13 x 13 cm. The order of the sunken reliefs was randomized between subjects. A schematic graph of the sunken reliefs is displayed in Fig. 3. After HE, the opaque screen was removed so the participants could move freely without any obstructions during RI. Participants’ eyes remained open during this experimental phase. In the study by Grunwald et al. (2014), the RI lasted 5 minutes, so it would be comparable in length to the other experimental phases—to make sure that the occurrence of sFST during the RI and their possible effect on EEG was not due to chance. As expected, the authors found significantly more sFST during the RI than during the other experimental phases. For this reason, the duration of the RI was extended to 14 minutes in the present study. During the following reproduction period, participants were to draw the structure of the sunken reliefs on a sheet of paper to keep up the illusion of a memory task. After reproduction, the opaque screen was reinstalled and the next two reliefs were presented. In the study by Grunwald et al. (2014), a total of four experimental blocks were run. We reduced the total number of experimental blocks from four to two in order to prevent exhaustion effects.

### Technical devices

A 19-channel digital EEG was continuously recorded for all participants in a Faraday Cage during the whole experiment using Ag–AgCl electrodes at standard electrode positions (Fp1, Fp2, F7, F3, Fz, F4, F8, T3, C3, Cz, C4, T4, T5, T6, P3, Pz, P4, O1, O2; reference: linked earlobes; International10–20 system (Jasper, 1958)). Movements of the eyes were monitored by horizontal (HEOG) and vertical (VEOG) electrodes. Electrical impedance was kept below 5 kΏ, sampling rate was 256 Hz. Facial self-touch movements and skin contact durations were measured via EMG (two electrodes placed on the dorsal sides of both the left and right forearm above m. extensor carpi ulnaris) and analogous, tri-axial acceleration sensors (ADXL335; attached to the wrists of the participants). The whole experiment was videotaped through a one-way mirror. The recording system (IT-med GmbH, Germany) allowed for parallel, synchronized recordings of EEG, EMG, acceleration sensors, and videos of the whole experimental session.

### Data analysis

The present study examined spontaneously occurring self-touches of the face in a study design with controlled trials. To define the type of sFST even more strictly, all self-touches of the hair, head, neck and ears were excluded as well as all sFST with obvious instrumental value (yawning, scratching, nose picking, etc.). Only sFST during RI were analyzed in the present study.

To prepare the data for analyses, EEG recordings were manually marked according to the different phases of the experiment (start – end baseline, start – end HE), artificial events (e.g., body or head movements) and sFST (start – end arm movement and start – end skin contact). The data of the acceleration sensors, EMG and the video recording were used as criteria for the markings. When a sFST was observed in the video, the traces of the accelerometers as well as the EMG were inspected for a visible slope that allowed us to precisely mark the beginning or end of a movement.

Preparation and segmentation of EEG data, ocular correction, artifact rejection, and subsequent calculations of the mean spectral power density were performed with an analytical EEG software package (Brain Vision 1.05, Brain Products, Munich, Germany). Data were filtered using IIR filter (zero phase shift Butterworth filter, low cutoff 0.5 Hz, high cutoff 70 Hz, order 2, notch filter 50 Hz). We used an ocular artifact correction (Gratton et al., 1983) and an automatic artifact rejection with an amplitude criterion of ±80 μV. We performed a spectral analysis of each artifact-free EEG segment using a Fast Fourier Transformation (FFT), after applying a 10% Hanning window. Resolution was set to 0.5 Hz (512 points using zero-padding). Mean spectral absolute power (μV^2^) was calculated as the mean amplitude of the spectral lines of the EEG bands (delta: 0.5-4.0 Hz; theta: 4.0–8.0 Hz; alpha: 8.0–13.0 Hz; beta: 13.0–24.0 Hz; gamma: 24.0–49.0 Hz). The following phases were used to calculate the EEG spectral power: Artifact-free EEG segments of the first experimental resting period (3 min, eyes open; EEG with 256 data points per segment) were used to analyze the spectral power at baseline. To analyze the artifact-free spectral power after haptic exploration (aHE), the first 3 EEG segments (3 s with 256 data points each) after the participants ceased exploration, were used. For the detailed analysis of sFST, the following epochs of sFST were segmented: Periods of 3 s before the start (b-sFST) and 3 s after the end (a-sFST) of arm movements as well as 500 ms at the start (c-start) and 500 ms at the end (c-end) of finger-face skin contacts were used to calculate the artifact-free spectral power.

To analyze frequency-specific changes in the continuous EEG, the mean spectral power parameters per channel, participant, and experimental phase were used. For statistical comparisons between spectral power per band and channel, nonparametric Wilcoxon signed-rank tests with adjusted Bonferroni-corrected alpha level (0.05/19, *p*_crit_ = 0.002) were used. Effect sizes for the Wilcoxon signed-rank tests were calculated as r = z/√N, where z is the z-score produced by Wilcoxon signed-rank test and N is the sample size. An effect size score of 0.1 indicates a small, 0.3 a medium, and ≥0.5 a large effect (Fritz et al., 2012). For comparisons of group frequencies Binomial tests were conducted. Independent *t*-tests was used for independent group comparisons. All statistical analyses were conducted using SPSS for Windows (version 25.0).

Probability Maps (Inhouse Software) were used to illustrate the topographic distribution of statistical test results. For this purpose, the *p*-values of the error probabilities per frequency range were displayed in graphical form. Empty (□) or filled squares (■) were used for each individual comparison per channel to mark the significant differences between two periods. An empty square represents a decrease of the spectral power, a filled square corresponds to an increase of the spectral power between two periods The different sizes of the squares represent the strength of the significance. Accordingly, a larger square indicates a higher significance. If a result did not reach the significance value *p* ≤ 0.05, it was displayed as a circle. Empty circles (○) indicate the tendency of a decrease and filled circles (●) indicate the tendency of an increase. The data of the current study are available from the corresponding author upon request.

## Results

### Descriptive statistics

Among the 44 participants who performed sFST during the RI, an average of M = 4.52 (SD = 3.49) sFST per participant were observed. The mean skin contact duration of sFST was M = 2.67 seconds (SD = 3.97). The number of individual sFST during the RI ranged from 1 to 14 sFST. Across the 44 participants, significantly more sFST were performed during the RI (sum = 199) than during all other experimental phases combined (sum = 108; Binomial test *p* < 0.001). Moreover, significantly more sFST occurred during the presentation of distracting sounds (sum = 136) than during the sound-free phases (sum = 63; Binomial test *p* < 0.001). After 36 sFST were rejected due to artifacts, a total of 163 sFST were included in the EEG analyses. Rejected sFST did not differ from included sFST in terms of skin contact duration (t(197) = −647, *p* = 0.518).

### Hypotheses 1a-c, replication of previous study results

#### Hypothesis 1a: Spectral EEG power increases between baseline and after haptic exploration (aHE)

**Fig. 4:**
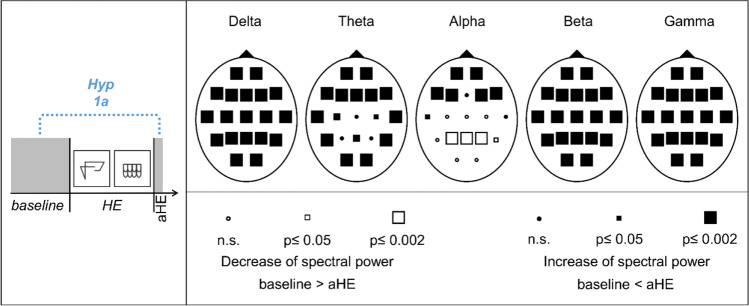
Schematic representation of hypothesis 1a (Hyp 1a) and Probability Maps for spectral EEG power comparisons between baseline and after haptic exploration (aHE). Results of nonparametric Wilcoxon-tests per channel and frequency band

Comparisons of the EEG at rest (baseline) and aHE showed significant increases of the spectral power above all electrodes for the delta, theta (except Cz, P3, P4), beta (except Pz), and gamma bands (Fig. 4). Alpha power increased above frontal electrodes and decreased above parietal regions. Effect sizes of the significant spectral power changes were medium to large (r = 0.4–0.9). Corresponding statistical values of the Wilcoxon tests and effect sizes are presented in Supplementary Table S1.

#### Hypothesis 1b: Spectral EEG power decreases between after haptic exploration (aHE) and before sFST (b-sFST)

**Fig. 5:**
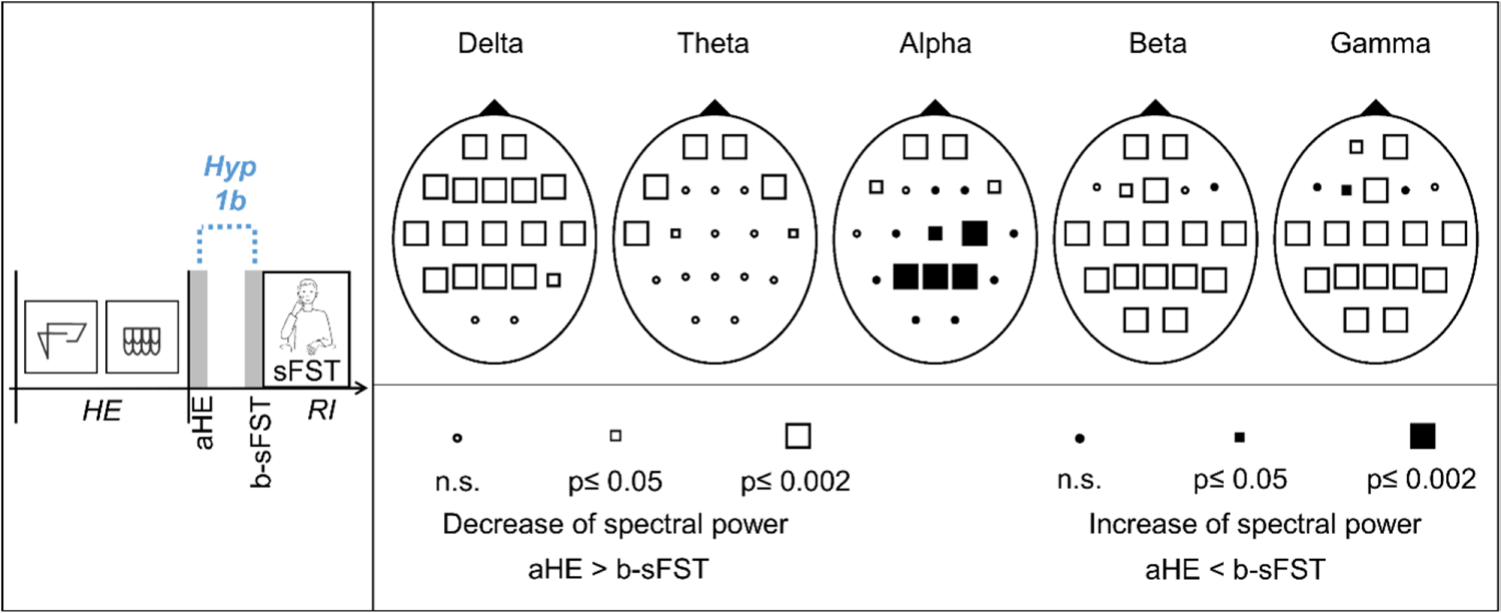
Schematic representation of hypothesis 1b (Hyp 1b) and Probability Maps for spectral EEG power comparisons between after haptic exploration (aHE) and before spontaneous facial self-touches (b-sFST). Results of nonparametric Wilcoxon-tests per channel and frequency band. RI = Retention Interval

Comparisons of the EEG aHE and b-sFST showed significant decreases of the spectral power for all frequency bands. In addition, a significant increase was recorded in the alpha band (Fig. 5). Globally distributed decreases of spectral power were observed for the delta band (except O1, O2), the beta band (except F4, F7, F8), and the gamma band (F3, F4, F7, F8). The theta power mainly decreased above frontal and temporal regions. In the alpha band, there was a significant decrease in spectral power above frontal areas, whereas there was a significant increase above parietal regions. Effect sizes of the significant spectral power changes were medium to large (r = 0.3–0.8). Corresponding statistical values of the Wilcoxon tests and effect sizes are presented in Supplementary Table S2.

#### Hypothesis 1c: Spectral EEG power increases between before sFST (b-sFST) and after sFST (a-sFST)

**Fig. 6:**
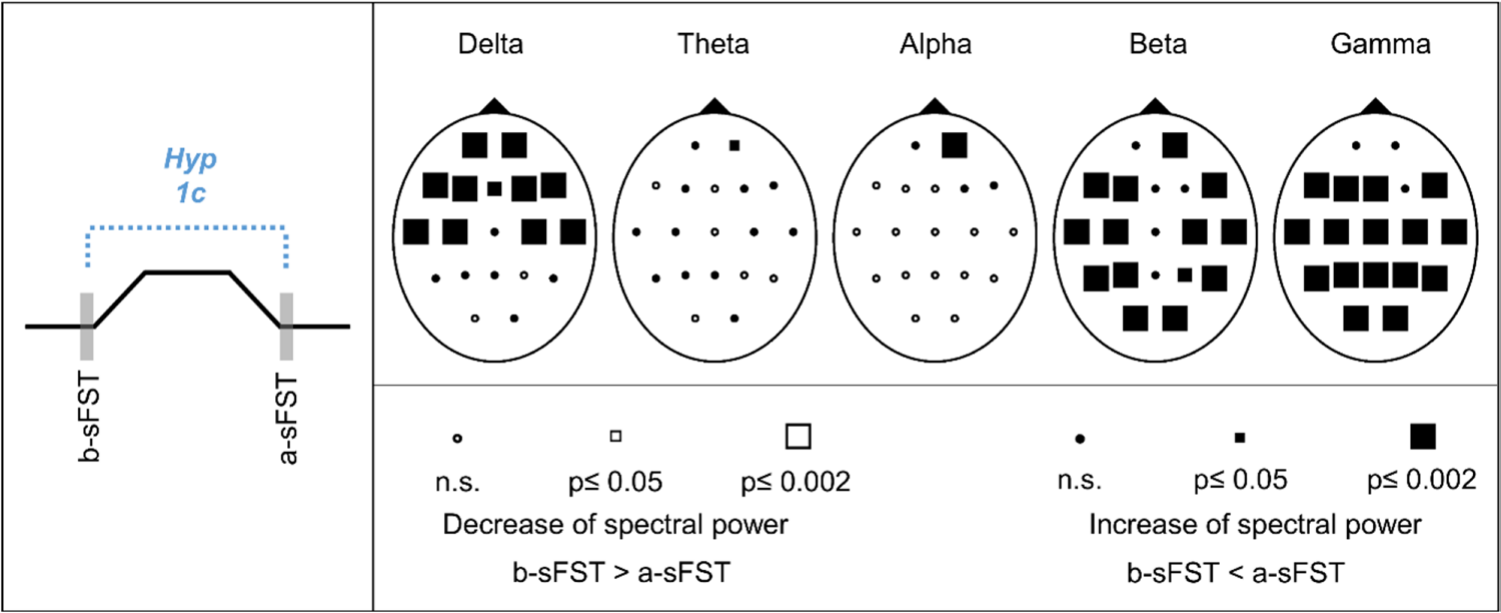
Schematic representation of hypothesis 1c (Hyp 1c) and Probability Maps for spectral EEG power comparisons between before spontaneous facial self-touches (b-SFST) and after sFST (a-sFST). Results of nonparametric Wilcoxon-tests per channel and frequency band

Comparisons of the EEG b-sFST and a-sFST showed significant increases of the spectral power for all frequency bands (Fig. 6). The spectral power of delta frequency showed significant increases above frontocentral and temporal regions. Increases of the theta and alpha band were observed over right prefrontal electrodes. However, the significant changes in theta and alpha did not reach the critical Bonferroni value of *p*_crit_ = 0.002. Bilateral increases over the whole cortex were observed for beta (excluding midline electrodes) and gamma (excluding Fp1, Fp2, F4). Effect sizes of the significant spectral power changes were medium to large (r = 0.3–0.7). Corresponding statistical values of the Wilcoxon tests and effect sizes are presented in Supplementary Table S3.

### Hypotheses 2a-c: Neurophysiological changes during the skin contact of spontaneous facial self-touches

#### Hypothesis 2a: Spectral EEG power changes between before sFST (b-sFST) and start of skin contact (c-start)

**Fig. 7:**
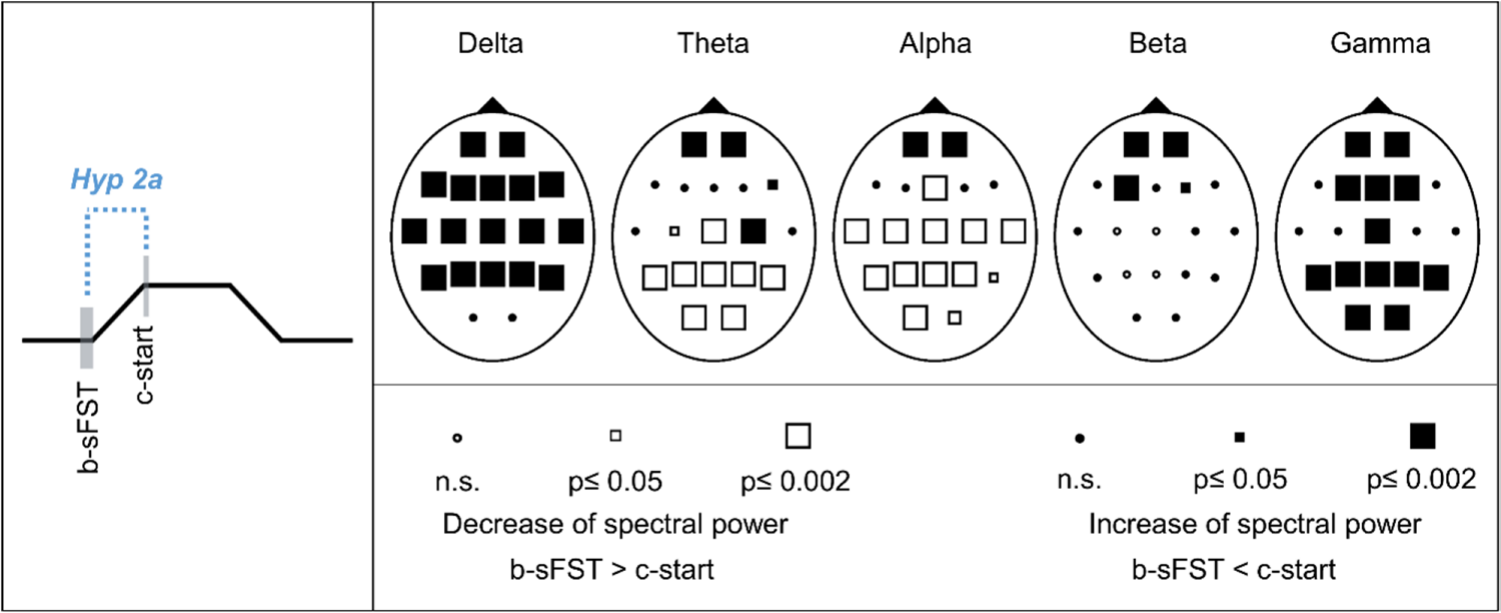
Schematic representation of hypothesis 2a (Hyp 2a) and Probability Maps for spectral EEG power comparisons between before spontaneous facial self-touches (b-sFST) and start of skin contact (c-start). Results of nonparametric Wilcoxon-tests per channel and frequency band

Comparisons of the EEG b-sFST and c-start showed significant increases of the spectral power for all frequency bands and additionally significant decreases in the theta and alpha band (Fig. 7). Delta power increased over the whole cortex (excluding O1, O2). Theta, alpha, and beta mainly increased above prefrontal and frontal regions. The spectral power of gamma frequency showed significant increases over the whole cortex (excluding F7, F8, T3, T4, C3, C4). Significant decreases occurred above parietal and occipital regions in the theta band and above centroparietal, temporal, and occipital regions in the alpha band. Effect sizes of the significant spectral power changes were medium to large (r = 0.3–0.8). Corresponding statistical values of the Wilcoxon tests and effect sizes are presented in Supplementary Table S4.

#### Hypothesis 2b: Spectral EEG power changes between start of skin contact (c-start) and end of skin contact (c-end)

**Fig. 8:**
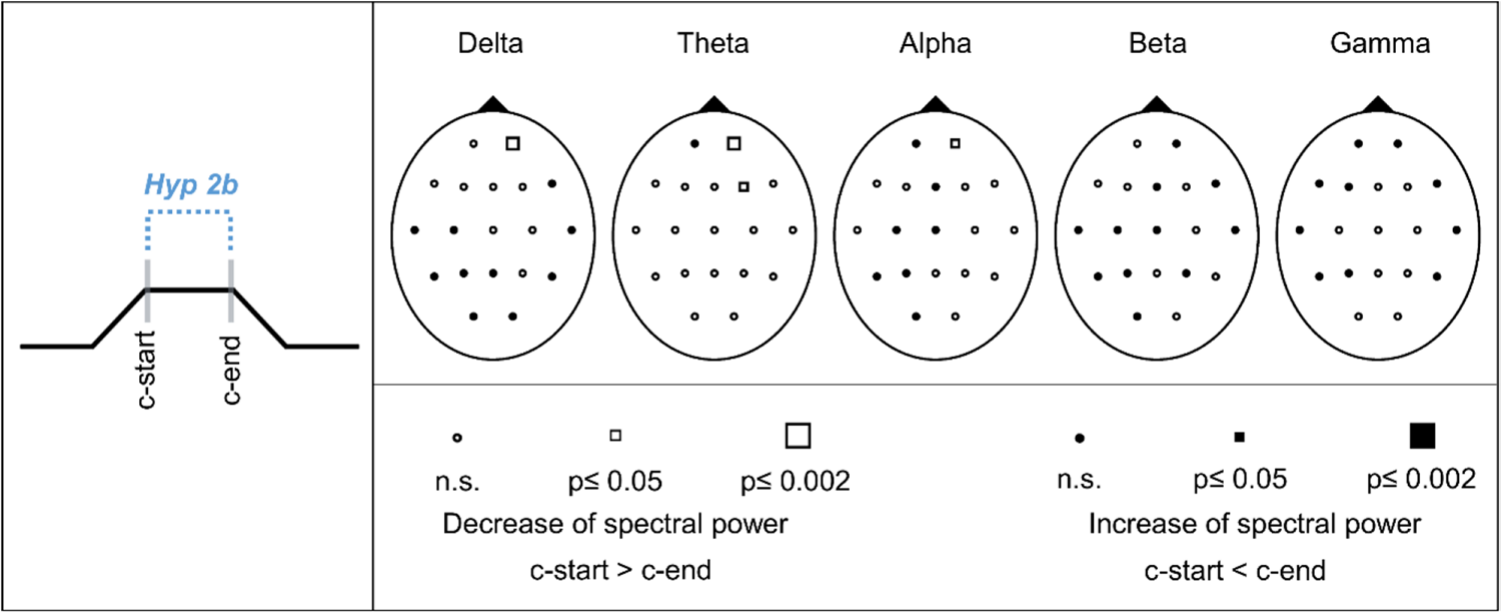
Schematic representation of hypothesis 2b (Hyp 2b) and Probability Maps for spectral EEG power comparisons between the start of skin contact (c-start) and the end of skin contact (c-end). Results of nonparametric Wilcoxon-tests per channel and frequency band

Comparisons of the EEG c-start and c-end showed no significant changes of the spectral power for any frequency band (Fig. 8). Significant decreases were observed in delta (Fp2), theta (Fp2, F4), and alpha (Fp2). However, these results did not reach the critical Bonferroni value of *p*_crit_ = 0.002. Corresponding statistical values of the Wilcoxon tests and effect sizes are presented in Supplementary Table S5.

#### Hypothesis 2c: Spectral EEG power changes between end of skin contact (c-end) and after sFST (a-sFST)

**Fig. 9:**
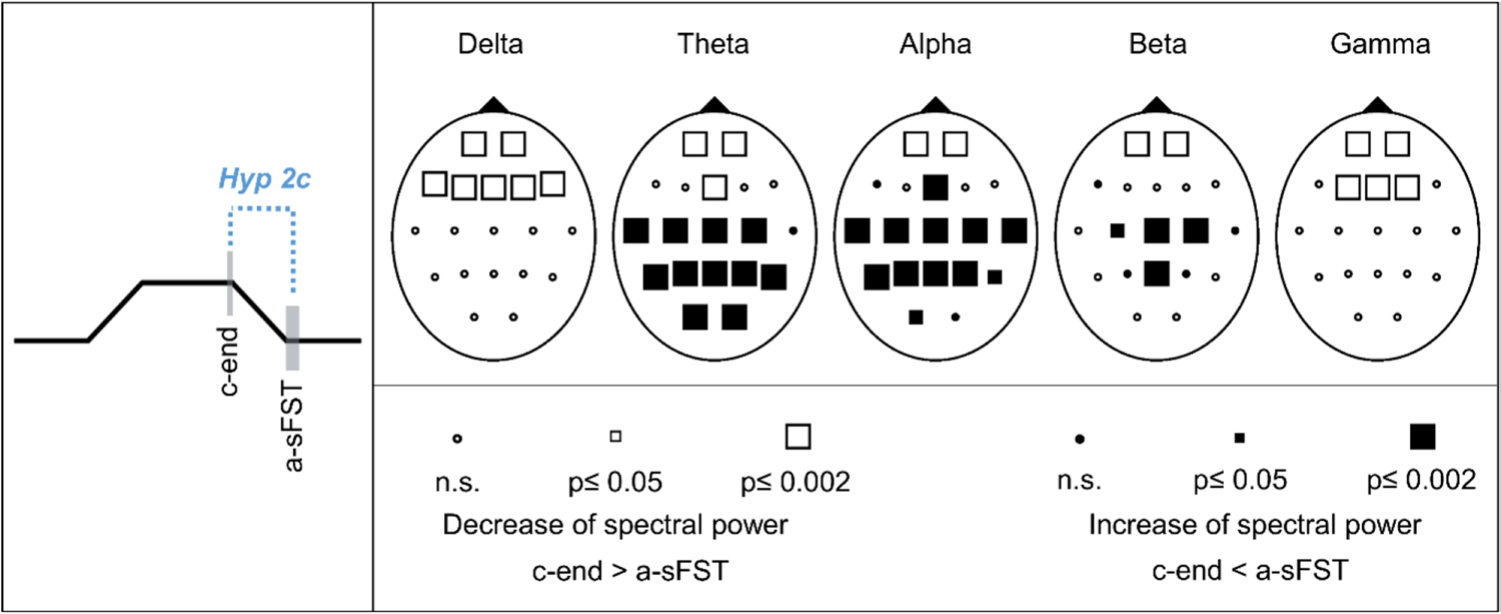
Schematic representation of hypothesis 2c (Hyp 2c) and Probability Maps for spectral EEG power comparisons between end of skin contact (c-end) and after spontaneous facial self-touches (a-sFST). Results of nonparametric Wilcoxon-tests per channel and frequency band

Comparisons of the EEG c-end and a-sFST showed significant decreases of the spectral power for all frequency bands above prefrontal and frontal regions (Fig. 9). Additionally, theta and alpha power increased above centroparietal, temporal and occipital regions. Significant increases of the beta band were observed over frontoparietal electrodes. Effect sizes of the significant spectral power changes were medium to large (r = 0.3–0.7). Corresponding statistical values of the Wilcoxon tests and effect sizes are presented in Supplementary Table S6.

Exemplary for the predominant EEG changes before, during, and after the skin contact phase of sFST, the mean spectral power (box plots) of the electrodes F3, Cz, P4, and O1 is depicted in Supplementary Figs. S2-S6 for all frequency bands.

## Discussion

The present study was able to replicate the results of our own previous research on neurophysiological mechanisms of spontaneous facial self-touches (sFST) in a larger sample. In accordance with the results of Grunwald et al. (2014), it was reconfirmed that neurophysiological changes occurred before and after sFST that indicate brain regulatory processes. Moreover, we found that these regulatory effects were activated in the first milliseconds of the execution of sFST. Within the discussion, we will particularly address the results that differ from those found by Grunwald et al. (2014).

### Spontaneous facial self-touches may represent working memory and attentional processes

In accordance with hypothesis 1a, significant increases in the delta, theta, beta, and gamma frequency bands were observed after haptic exploration of two sunken reliefs (aHE) compared with a 3-minute baseline. These increases may indicate encoding processes of bottom-up information as well as a high memory load as a consequence of the working memory task (Deiber et al., 2007; Friese et al., 2013; Harmony et al., 2004; Klimesch et al., 1996; Mölle et al., 2002; Onton et al., 2005; Weiss & Rappelsberger, 2000). Studies found focused attention to internal processing and mental effort to be associated with increases in spectral delta (Fernandez et al., 2002; Fernández et al., 1995; Harmony et al., 2004) and theta power (Deiber et al., 2007; Gevins et al., 1997; Jensen & Tesche, 2002). Increases in the gamma band have been discussed in association with the maintenance of object representations held in memory (Mainy et al., 2007; Tallon-Baudry et al., 1999). In line with hypothesis 1b, significant decreases in spectral delta, theta, and gamma power were observed shortly before the self-touch. The decrease in spectral power shortly before sFST may indicate impaired attention to the maintaining processes during the retention interval (RI). Research findings suggest that distracting sounds divert the focus of attention from the memory that should be maintained in a working memory task (Bell et al., 2010; Campbell et al., 2002). According to Grunwald et al. (2014), sFST are performed as a consequence of such interference with maintenance processes. This assumption is supported by the finding that significantly more sFST occurred during the presentation of distracting sounds than in the silences between sounds.

Grunwald et al. (2014) speculated that spectral power increases a-sFST compared to b-sFST may represent processes of working memory maintenance. As expected in hypothesis 1c, significant increases a-sFST were observed that may indicate such brain regulatory functions. For one, delta power increased a-sFST above anterior regions. Such heightened delta power has been associated with processes related to concentration and sustained attention during the retention of information in working memory (Fernandez et al., 2002; Harmony et al., 2004). Furthermore, a-sFST widespread increases of spectral power occurred in the gamma band. Increases in gamma have been associated with the maintenance of sensory working memory representations (Roux & Uhlhaas, 2014). In addition, gamma oscillations are thought to play a special role in integrating multiple feature-specific information into coherent object representations, because stronger activity increases have been found for more complex stimuli (e.g., shapes) than for simple stimuli (e.g., color) (Herrmann et al., 2004; Honkanen et al., 2015). It has been hypothesized that complex stimuli are not processed in a single cortical location but may require distributed neural networks that are spread across several different cortical areas (Christophel et al., 2017; Fuster, 2000). The distributed increase in spectral gamma power a-sFST might reflect the maintenance of a complex object representation in working memory. The haptic stimuli used in the present experimental setting were complex geometric forms and had a variety of features, including material texture, relief (depth, width, start and end points) and orientation. This multitude of features had to be combined into a coherent object presentation for two stimuli. The EEG pattern b-sFST supports the assumption of working memory activation, because gamma power decreased shortly before the occurrence of sFST. Tallon-Baudry and colleagues observed an association between the decreasing performance of the participants and the decreasing energy of spectral gamma power with increasing delay time during a working memory task (Tallon-Baudry et al., 1999). Furthermore, the prefrontal increase in the beta power a-sFST may be related to (re)activation of working memory content (Spitzer & Haegens, 2017). Studies on working memory tasks have found prefrontal increases in the beta band at the end of retention phases when memory content was endogenously "refreshed" in preparation for the imminent comparison task (Spitzer et al., 2010) or when participants were explicitly (retro)-cued to update task-relevant memory contents (Spitzer & Blankenburg, 2011). To test the assumption that the performance of sFST is associated with the maintenance of working memory content, future studies should investigate which behavioral as well as neurophysiological effects are associated with suppression of sFST during working memory maintenance.

### The involvement of spontaneous facial self-touches in emotional processes

We observed decreases in spectral theta and alpha power above prefrontal and frontal regions b-sFST. According to Grunwald et al. (2014), neurophysiological decreases in spectral theta power b-sFST may indicate internal contemplation about distracted attention and the fading memory which in turn may have led to emotional reactions. Moreover, the content of the distracting sounds (e.g. baby crying, explosion) may itself have elicited an emotional response. Grissmann et al. (2017) found that interfering stimuli with negative valence during a working memory task led to a decrease in performance and decreased frontal theta activity that could not be explained by additional working memory load. Likewise, the present results showed a decrease in prefrontal theta power b-sFST, which could be due to the distracting sounds.

Furthermore, studies have discussed an association of active emotion regulation, e.g., cognitive reappraisal, with prefrontal power increases in theta (Ertl et al., 2013) and alpha bands (Jackson et al., 2003; Tortella-Feliu et al., 2014). Automatic emotion regulation, in which the emotional meaning of a stimulus is not the explicit focus of the task to be performed, also was mentioned in association with activations in prefrontal areas (Ochsner et al., 2012; Rive et al., 2013). In the present study, the theta and alpha frequency bands showed a decrease in spectral band power above prefrontal regions b-sFST, followed by a power increase above the same areas a-sFST. Although the power increase in the frequency bands a-sFST did not reach the critical Bonferroni criterion, the EEG pattern supports the hypothesis that sFST may be involved in emotional regulation processes, due to the negative valence as well as the distraction effect caused by the sounds. Recent studies have shown a positive association between trait anxiety and the number of sFST (Carrillo-Díaz et al., 2020; Carrillo-Díaz et al., 2021). Because touching one’s own face is associated with the transmission of pathogens, a number of researchers addressed the need to reduce face-touching behaviors (Chen et al., 2020; Lucas et al., 2020; Senthilkumaran et al., 2020). However, following the interpretation that sFST serve emotion regulatory functions, it may be inadvisable—particularly for anxious people—to suppress this behavior. Future studies should investigate whether neurophysiological parameters of sFST differ between individuals with high- and low-trait anxiety and assess the consequences of suppressing self-touch behavior in these groups.

### Spontaneously touching the own face—a simple behavior with complex causes

It should be emphasized that the underlying trigger mechanisms of sFST are presumably complex in nature. Test subjects did not show increased numbers of sFST when listening to unpleasant sounds from the IADS-2 compared with an unchallenging quiet situation (Grunwald et al., 2014). In another pre-study, participants completed a complex haptic memory task without additional distracting sounds. Again, the test subjects did not show increased numbers of sFST. In the present experiment, information had to be maintained in working memory while distracting sounds were presented. The combination of these two demands resulted in an increased number of sFST. Several studies have found evidence for cognitive-emotional integration processes in the prefrontal cortex (Pessoa, 2008). For example, Perlstein et al. (2002) found activity changes in the prefrontal cortex during working memory tasks in which participants had to remember emotional pictures. These changes were not observed when the emotional pictures were presented without participants being asked to retain information in memory (Perlstein et al., 2002). The prefrontal cortex also has been discussed in association with the emergence of conflict and cognitive control in challenging situations (Botvinick et al., 2001). Conflict monitoring might represent one aspect of a more general monitoring function that detects internal states, signaling a need to intensify or redirect attention or control (Botvinick et al., 2004). The spectral power increases of theta and alpha a-sFST above prefrontal areas could indicate the activation of cognitive processes and not merely reflect emotional regulation processes. However, based on the available data, it is not possible to decide between these possible interpretations and additional work is needed to clarify this issue. To assess whether the observed prefrontal spectral power increases a-sFST are due to either emotional processes or cognitive demands, future studies should use distracting stimuli without emotional valence. Furthermore, it should be tested whether sFST are associated with similar neurophysiological changes when experiments on attention and conflict control (e.g., Stroop task or flanker task) are conducted without additional working memory load.

### EEG-pattern of the alpha frequency band during delayed memory task

The present results on the alpha band power differ considerably from those of Grunwald et al. (2014). Contrary to hypotheses 1a-c, there were characteristic changes in spectral alpha power before and after the performance of sFST. The observed parietal decreases aHE compared with baseline may indicate the high memory load as a consequence of memory encoding (Babu Henry Samuel et al., 2018; Sauseng et al., 2005). The increase in frontal alpha power aHE can be interpreted in the context of top-down control mechanisms and inhibition processes that are activated at early stages of information processing (Klimesch et al., 2007; Sauseng et al., 2005; Zhang & Ding, 2010). The centroparietal increases in spectral alpha power b-sFST might reflect a sensory inhibition mechanism in response to the presentation of the distracting sounds to avoid interference with the working memory trace during RI (Babu Henry Samuel et al., 2018; Bonnefond & Jensen, 2012; Tuladhar et al., 2007). In addition, the long duration of the RI in the present study (14 min) may have led to other effects that were not evident in the preliminary study by Grunwald et al. (2014), in which the RI lasted only 5 min. We speculate that the use of longer retention times may be more representative for everyday challenges. Remembering relevant information for several minutes may be difficult due to distracting sensory input as well as mind wandering. In line with that, Baldwin et al. (2017) found an increase in parietal alpha power associated with mind wandering. However, which internal processes led to a decrease in spectral alpha power above frontal regions b-sFST remains unclear. While some studies observed a decrease in frontal alpha power during high memory load (Crespo-Garcia et al., 2013; Stipacek et al., 2003), other authors found opposite results (Michels et al., 2010; Sauseng et al., 2005). The different results may indicate that different regions within the frontal cortex are activated depending on the mental operations required in the different working memory tasks (Crespo-Garcia et al., 2013). The longer duration of the RI in the present study might have led to a higher memory load—accompanied by corresponding changes in the alpha band—than in the preliminary study of Grunwald et al. (2014), in which the RI lasted only 5 minutes. The divergent findings in the alpha band also may be related to the extended number of participants compared with the study by Grunwald and colleagues.

### Regulatory processes of brief spontaneous facial self-touches are activated in the first milliseconds of execution

As expected in hypothesis 2a and 2c, the results showed significant changes in all analyzed frequency bands at the beginning and at the of the skin contact phase of sFST, respectively. At the beginning of the skin contact of sFST, increases in the delta, beta, and gamma band were observed above those cortical areas previously discussed in the context of regulatory processes during working memory demands (cf., hypothesis 1c). Moreover, the theta and alpha band showed significant increases above those areas previously discussed in the context of conflict monitoring and emotion regulation processes (cf., hypothesis 1c). Contrary to hypothesis 2b, we did not find significant spectral power changes during the skin contact phase of sFST—instead, the spectral power remained at a constant level during skin contact. At the end of sFST, the spectral power decreased again above those regions where it had increased at the beginning of the skin contact phase. Although the spectral power decreased at the end of sFST, the spectral power after sFST was overall still higher than before sFST. These dynamic spectral power changes over the course of sFST (Supplementary Figs. S2-S6) indicate that the presumed cognitive and emotional regulatory processes were activated at the beginning of the skin contact of sFST and continued for the duration of skin contact.

The skin contact phases of sFST in the present study were of short duration (M = 2.67 s; SD = 3.97). Decades ago, Freedman discussed that brief touch events (3 s or less) differ from continuous sFST (in some instances more than 100 s); not only in their duration, but also in their function (Freedman, 1972). A recent review on sFST also found that the average duration of sFST varied from less than 3 s to more than 10 s (Spille et al., 2021). Assuming that brief sFST are a direct regulatory response to emotionally and cognitively disturbing situations, the question arises whether continuous sFST with longer skin contact phases serve different functions than brief sFST and, accordingly, show different neurophysiological patterns.

In addition to the observed spectral power changes during sFST associated with regulatory processes, we observed significant power changes in the theta and alpha bands above posterior regions. It is unclear which processes led to a decrease in posterior theta and alpha power at the beginning, followed by an increase above the same regions at the end of sFST. As these spectral power changes occurred above areas of motor and sensory cortex, the observed posterior changes may be related to motor (movement of the hand toward the face) or sensory (tactile stimulation by skin contact between finger and face) aspects of sFST. Because we limited the present analysis to the skin contact phase, it cannot be clarified whether motor aspects, sensory skin contact, or an interaction of both led to the observed changes above posterior regions in theta and alpha. In contrast, the increases in spectral beta power above sensorimotor areas a-sFST are consistent with the findings of Grunwald et al. (2014) and may reflect the motor aspects of movement execution of sFST and indicate a post movement beta rebound (Alegre et al., 2004; Kilavik et al., 2013). In addition to the skin contact phase, future studies should investigate spectral power changes that occur during the movement phase of sFST.

### Limitations and future directions

In this study, our goal was to examine the spectral power changes that occur during spontaneous self-touches to the face. The experiment is characterized by a variety of independent variables, such as varying working memory load and distracting sounds with emotional content. This also is reflected in the multitude of EEG changes observed in all frequency bands before, during, and after sFST. On the one hand, the challenging experiment rather reflects everyday situations, as opposed to settings in which participants are not allowed to move and are required to perform: for example, stimulus-response tasks that are less complex. On the other hand, the present study setting increases the difficulty of interpreting the neurophysiological parameters, because the trigger mechanisms of sFST are probably complex. Therefore, limitations of the current study as well as recommendations for further work will be given in the following.

Studying spontaneous behaviors within controlled experimental trials is a difficult endeavor since spontaneous behaviors occur with individually varying frequency, are not strictly predictable, and may only be provoked to a limited extent in the context of an experiment. Therefore, neurobiological analysis of spontaneously occurring behavior is inherently limited with respect to the trials that can be obtained in laboratory studies and differs fundamentally from stimulus-response paradigms, in which the number of trials is determined before the experiment. In the present study, the participants performed an average of M = 4.52 (SD = 3.49) sFST. The comparatively small number of trials to be analyzed is an important limitation that also pertains to the investigation of other spontaneous behaviors, such as yawning, epileptic seizures, or lucid dreaming (Guggisberg et al., 2007; Sato et al., 2017; Voss et al., 2009, respectively). Despite these methodological limitations, given the large sample size and the total number of sFST trials, we consider the present data to be suitable for investigating sFST in a biologically representative and reliable manner. To further evaluate the reliability of the EEG measures, future studies could conduct the experimental procedure twice on the same sample to analyze the test-retest reliability of the spectral EEG parameters.

For a better understanding of internal and external trigger mechanisms and functions of sFST, future experimental procedures should make a clearer distinction between possible influencing factors. To address the emotion regulation hypothesis, subjectively experienced emotions should be rated by participants. Moreover, Schweizer et al. (2019) discussed that the positive or negative value to a healthy research participant will usually be relatively low for standardized affective stimuli. Therefore, valence and arousal of emotional stimuli need to be increased in future studies. To investigate the presumed function of sFST regarding attentional processes, different attention tasks should be conducted without applying additional working memory load. In terms of the regulation hypothesis of working memory processes, distractors without emotional valence should be applied. In the present study, we used a haptic working memory task during which EEG changes due to working memory load have been observed before (Grunwald et al., 1999, 2001; Grunwald et al., 2004; Grunwald et al., 2014). Nevertheless, it should be noted that, for example, visual or auditory working memory tasks are more established in EEG research than haptic memory tasks (Li Hegner et al., 2007). Following the hypothesis that sFST occur when maintenance processes are impaired by distractors, working memory tasks in other sensory modalities also should be applied and compared with the results of the present study. Regardless of the sensory modality used in a working memory task to investigate sFST, memory performance should be examined after the execution of sFST compared to a control situation in which sFST are prevented. Furthermore, to test the regulation hypothesis, other biological markers, such as autonomic activity or electrodermal activity, could be examined in addition to neurophysiological parameters.

With respect to the discussion of the risk of infection transmission by touching one's own face, future studies should take a closer look at the specific touched facial areas. In a recent review, Spille et al. (2021) found that most sFST are directed to the middle axis of the face. As the facial mucous membranes (eyes, nose, mouth) can get inoculated with bacteria from the fingertips (Rusin et al., 2002), it should be clarified whether sFST neurophysiologically differ depending on the executing hand (left- or right-handed movements) or touched area of the face. Future studies also should investigate whether body movements without skin contact that occur when using the same experimental setting are accompanied by similar neurophysiological changes as sFST.

Moreover, because sFST seem to occur more frequently than spontaneous touches of other body parts (Spille et al., 2021), it should be clarified whether other types of spontaneous self-touch show different neurophysiological patterns or have similar brain regulatory functions as sFST. A neuroimaging study by Boehme and colleagues recently investigated the processing of self-generated touch and touch by others at cortical levels (Boehme et al., 2019). However, unlike the work of these authors and other research addressing self-touch (Gentsch et al., 2015; Kilteni & Ehrsson, 2020), the present study investigated spontaneously occurring facial self-touches, as opposed to experimental conditions in which participants are instructed to actively touch themselves. Whether phenomena that occur in active self-touch, such as sensory attenuation (Kilteni & Ehrsson, 2020), similarly occur in sFST remains to be answered.

## Conclusions

Our results show that brief, spontaneous, facial self-touches are associated with neurophysiological changes indicative of internal regulatory processes. However, it remains unclear what exactly triggers sFST. In the present study, spectral power changes in the delta, theta, alpha, beta, and gamma frequency band were investigated, which occurred before, during, and after the execution of sFST and which were performed during the retention interval of a delayed haptic working memory task. There is evidence to suggest that changes in spectral delta and gamma power during the execution of sFST represent a refocusing of attention on the memory representations to be maintained. Prefrontal changes in theta and alpha power spectra may further indicate involvement in processes of emotional homeostasis. The results show that activations associated with regulatory processes occur in the first milliseconds of sFST, when the hand moves toward the face and touches the facial skin.

## Supplementary Information

ESM 1 (DOCX 3.24 kb)
