# Supplementary material for: Cognitive and emotional regulation processes of spontaneous facial self-touch are activated in the first milliseconds of touch: Replication of previous EEG findings and further insights
Source: Cogn Affect Behav Neurosci. 2022 Feb 19;22(5):984–1000. doi: 10.3758/s13415-022-00983-4 (PMC8857530; doi:10.3758/s13415-022-00983-4)
Supplement: Supplementary file 1 — (DOCX 3.24 kb) [file 13415_2022_983_MOESM1_ESM.docx]

**Supplementary material**

Description of the sound material:

The stimulus material consisted of a total of 60 sounds. 46 sounds were taken from the International Affective Digitized Sounds Database (2nd Edition; IADS-2). The stimuli are standardized, emotionally evocative sounds that cover a wide range of semantic categories. In a normative study by Bradley and colleagues, the affective valence of the stimuli was rated by a total of 100 participants on a 9-point rating scale (Bradley et al., 2007). Ratings were scored such that 9 represents a high rating (high pleasure) and 1 represents a low rating (low pleasure). We chose stimuli whose valence was rated as low pleasure (M = 3.21, SD = 1.07), e.g., baby crying, explosion or siren. Additionally, we chose another 14 sounds from a free audio database (https://freesound.org) that we judged as unpleasant, e.g., dentist drill or jackhammer. All sounds are available on the website of the Haptic Research Laboratory (https://haptiklabor.medizin.uni-leipzig.de) under “Research” > “Aversive Sounds for sFTG”.

Sound numbers IADS-2:

100, 105, 106, 115, 116, 130, 133, 251, 252, 261, 276, 277, 278, 279, 280, 285, 286, 287, 290, 291, 292, 310, 319, 322, 380, 403, 420, 422, 423, 424, 500, 501, 502, 600, 625, 626, 698, 699, 702, 706, 708, 709, 711, 712, 723, 730.


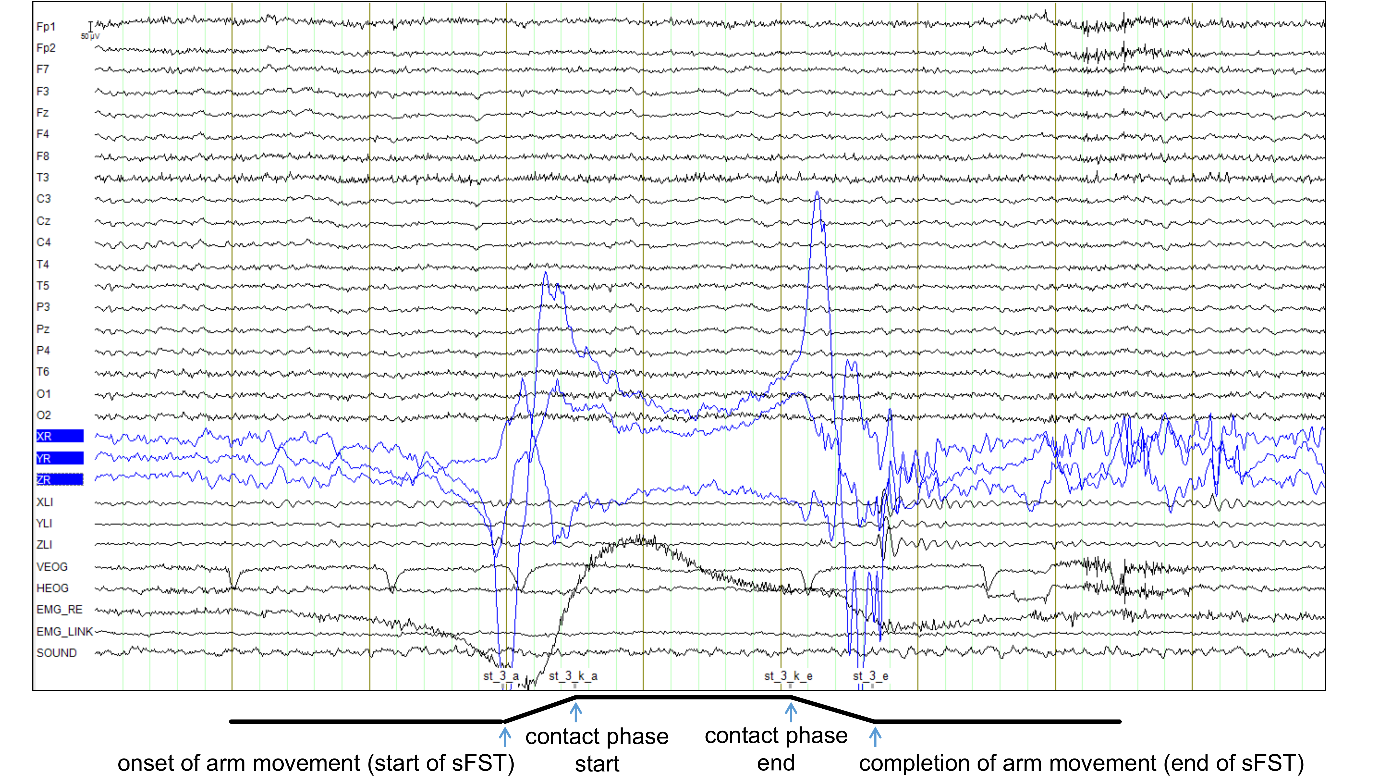


**Figure S1.** EEG segmentation of the different phases of a spontaneous facial self-touch (sFST) with markings of the start of sFST (onset of arm movement), skin contact phase (start and end) and end of sFST (completion of arm movement). Example of a typical segment with one right-handed sFST indicated. EEG channels (Fp1, Fp2, F7, F3, Fz, F4, F8, T3, C3, Cz, C4, T4, T5, P3, Pz, P4, T6, O1, O2), triaxial accelerometers of the right arm (XR, YR, ZR, three upper blue traces), the left arm (XLI, YLI, ZLI), EMG of the right arm (EMG_RE) and the left arm (EMG_LINK) as well as the sound track (SOUND) are displayed.


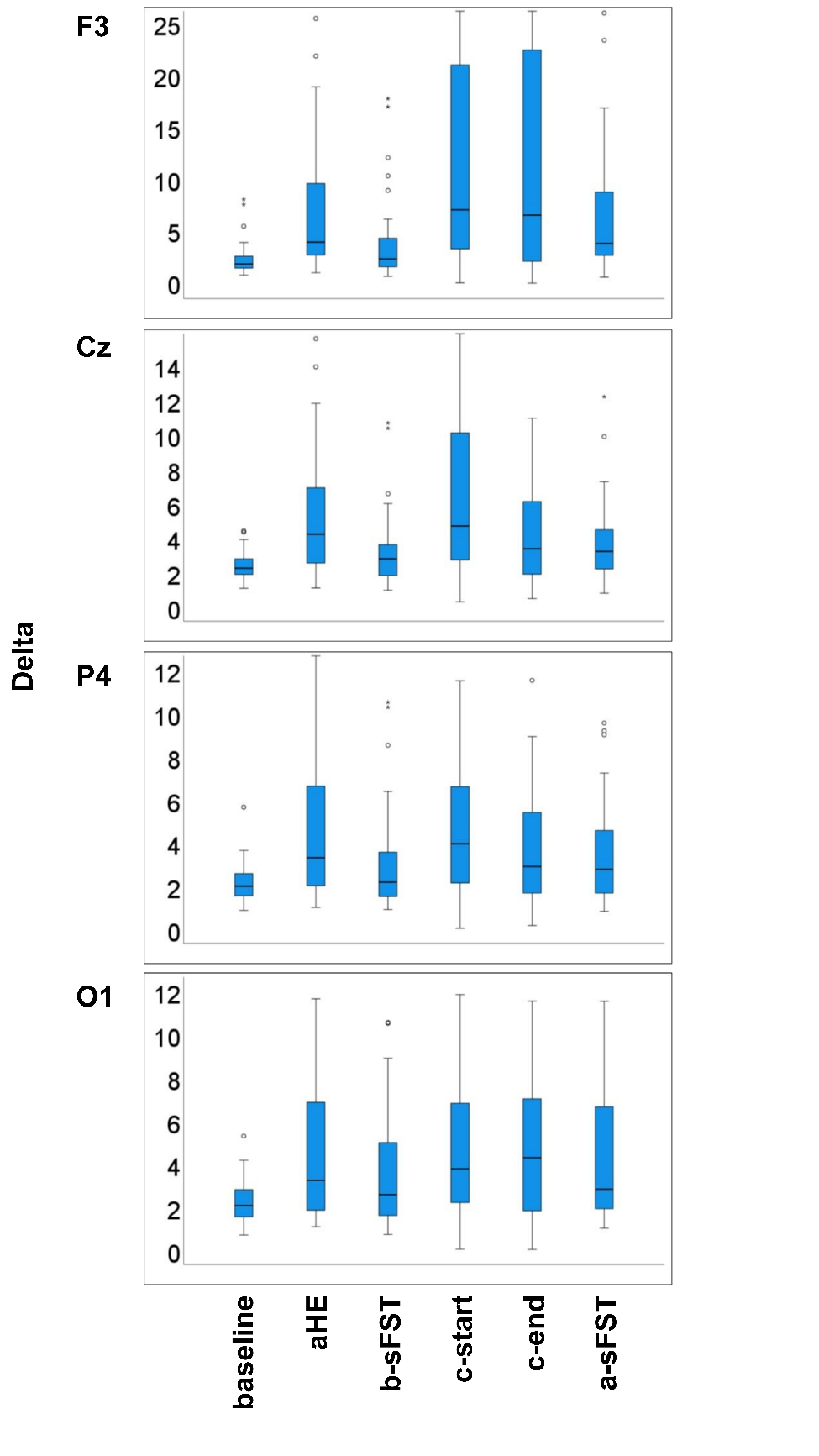


**Figure S2.** Box plots of mean spectral power (μV^2^) of the delta frequency band during the experimental periods: baseline, after haptic exploration (aHE), 3 s before spontaneous facial self-touch (b-sFST), first 500 ms of skin contact (c-start), last 500 ms of skin contact (c-end) and 3 s after spontaneous facial self-touch (a-sFST) for the electrodes F3, Cz, P4, O1.


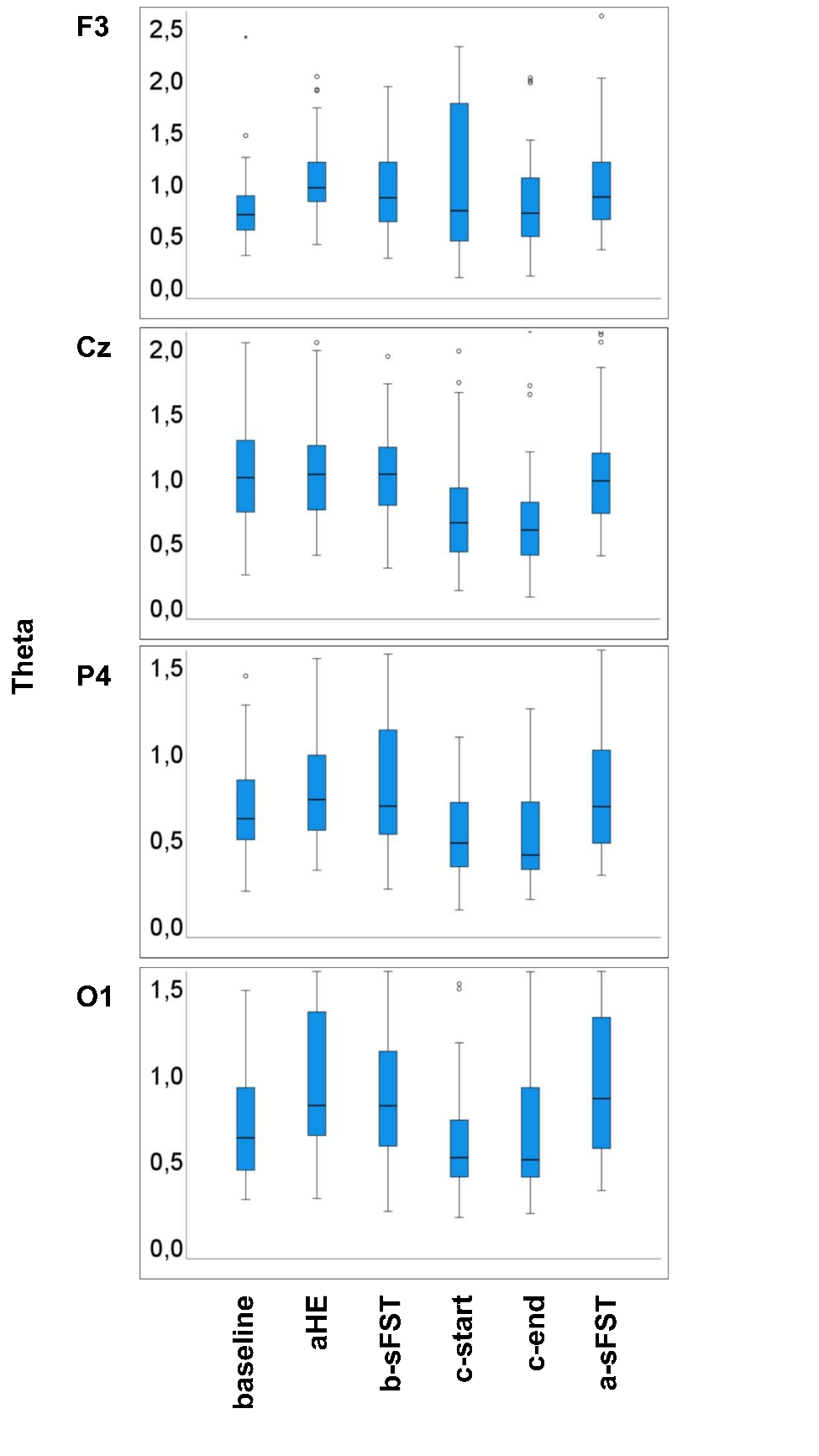


**Figure S3.** Box plots of mean spectral power (μV^2^) of the theta frequency band during the experimental periods: baseline, after haptic exploration (aHE), 3 s before spontaneous facial self-touch (b-sFST), first 500 ms of skin contact (c-start), last 500 ms of skin contact (c-end) and 3 s after spontaneous facial self-touch (a-sFST) for the electrodes F3, Cz, P4, O1.


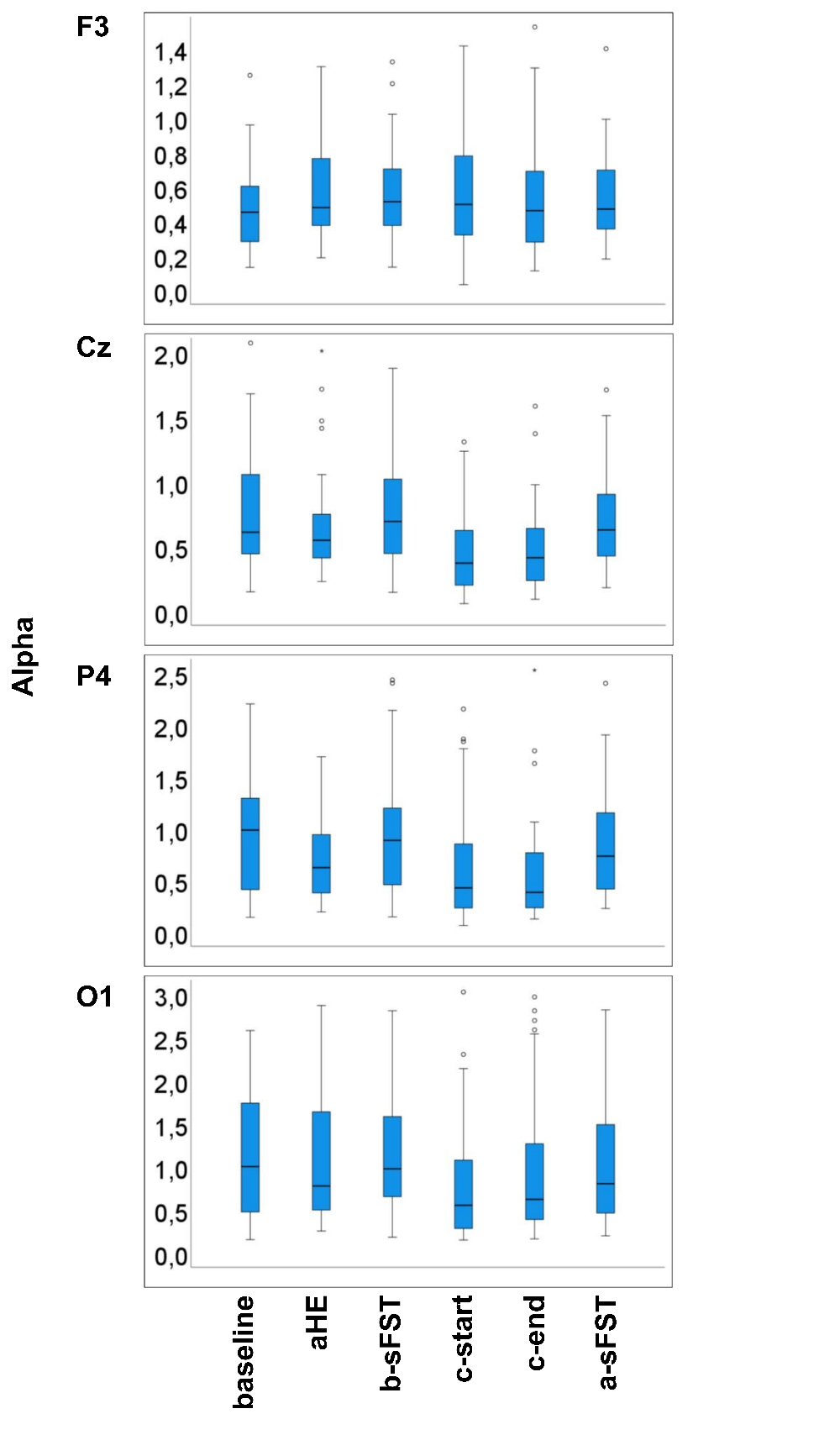


**Figure S4.** Box plots of mean spectral power (μV^2^) of the alpha frequency band during the experimental periods: baseline, after haptic exploration (aHE), 3 s before spontaneous facial self-touch (b-sFST), first 500 ms of skin contact (c-start), last 500 ms of skin contact (c-end) and 3 s after spontaneous facial self-touch (a-sFST) for the electrodes F3, Cz, P4, O1.


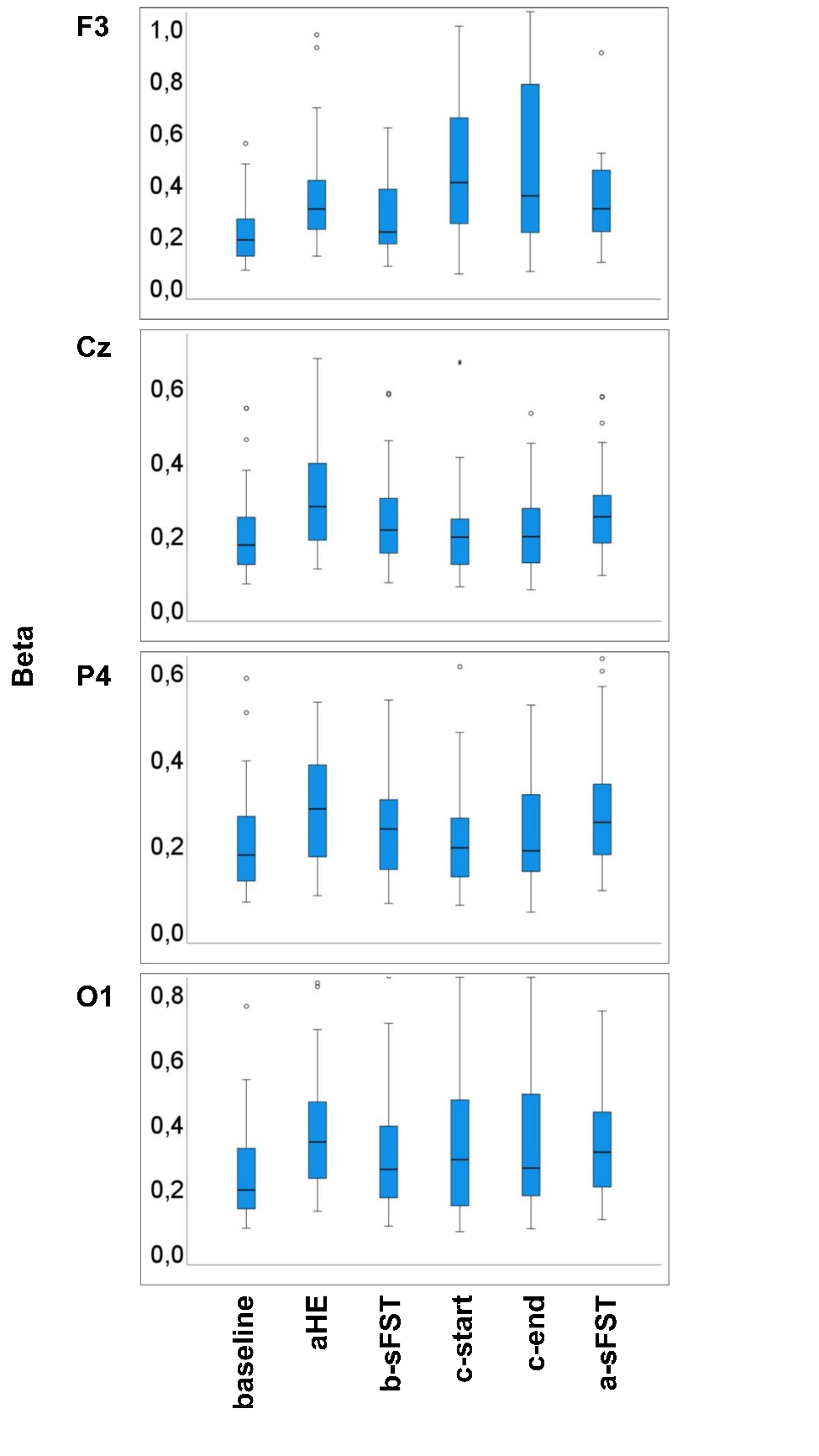


**Figure S5.** Box plots of mean spectral power (μV^2^) of the beta frequency band during the experimental periods: baseline, after haptic exploration (aHE), 3 s before spontaneous facial self-touch (b-sFST), first 500 ms of skin contact (c-start), last 500 ms of skin contact (c-end) and 3 s after spontaneous facial self-touch (a-sFST) for the electrodes F3, Cz, P4, O1.


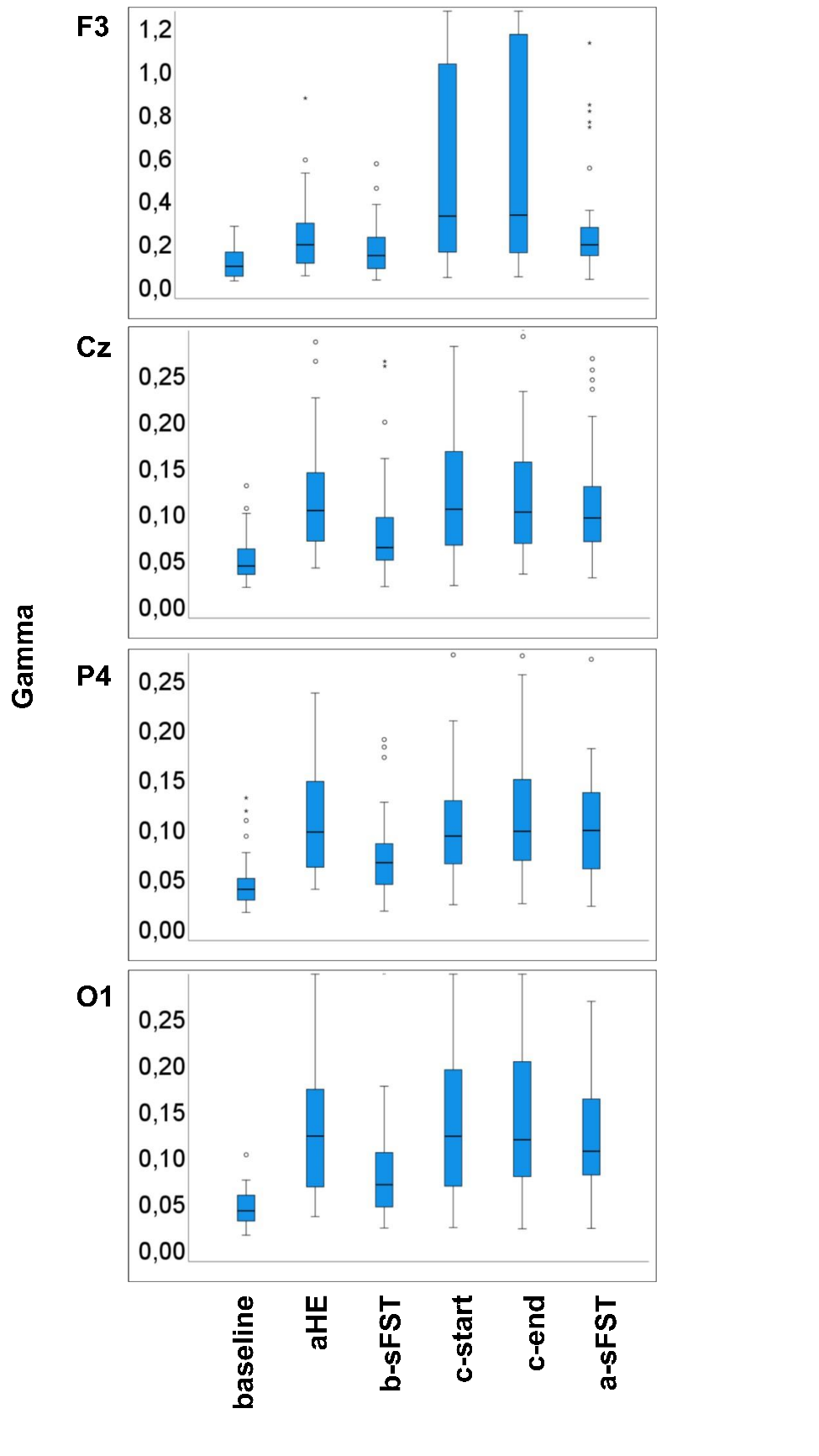


**Figure S6.** Box plots of mean spectral power (μV^2^) of the gamma frequency band during the experimental periods: baseline, after haptic exploration (aHE), 3 s before spontaneous facial self-touch (b-sFST), first 500 ms of skin contact (c-start), last 500 ms of skin contact (c-end) and 3 s after spontaneous facial self-touch (a-sFST) for the electrodes F3, Cz, P4, O1.

| Table S1. Hypothesis 1a: Spectral EEG power comparisons between baseline and after haptic exploration (aHE) | | | | | | | | | | | | | | | |
| --- | --- | --- | --- | --- | --- | --- | --- | --- | --- | --- | --- | --- | --- | --- | --- |
| EEG electrodes | Delta | | | Theta | | | Alpha | | | Beta | | | Gamma | |  |
|  | z | *p* | *r* | z | *p* | *r* | z | *p* | *r* | z | *p* | *r* | z | *p* | *r* |
| Fp1 | -4.633 | 0.000 | 0.7 | -5.695 | 0.000 | 0.9 | -5.730 | 0.000 | 0.9 | -5.252 | 0.000 | 0.8 | -4.901 | 0.000 | 0.7 |
| Fp2 | -4.365 | 0.000 | 0.7 | -5.438 | 0.000 | 0.8 | -5.683 | 0.000 | 0.9 | -5.567 | 0.000 | 0.8 | -4.633 | 0.000 | 0.7 |
| F7 | -5.100 | 0.000 | 0.8 | -5.718 | 0.000 | 0.9 | -4.878 | 0.000 | 0.7 | -5.380 | 0.000 | 0.8 | -4.365 | 0.000 | 0.7 |
| F3 | -4.563 | 0.000 | 0.7 | -4.843 | 0.000 | 0.7 | -2.778 | 0.005 | 0.4 | -5.217 | 0.000 | 0.8 | -4.785 | 0.000 | 0.7 |
| Fz | -5.007 | 0.000 | 0.8 | -2.883 | 0.004 | 0.4 | -0.829 | 0.407 | 0.1 | -5.438 | 0.000 | 0.8 | -5.485 | 0.000 | 0.8 |
| F4 | -4.820 | 0.000 | 0.7 | -3.804 | 0.000 | 0.6 | -2.521 | 0.012 | 0.4 | -5.228 | 0.000 | 0.8 | -4.225 | 0.000 | 0.6 |
| F8 | -4.796 | 0.000 | 0.7 | -5.520 | 0.000 | 0.8 | -4.575 | 0.000 | 0.7 | -4.505 | 0.000 | 0.7 | -3.466 | 0.001 | 0.5 |
| T3 | -5.182 | 0.000 | 0.8 | -4.820 | 0.000 | 0.7 | -2.019 | 0.043 | 0.3 | -4.470 | 0.000 | 0.7 | -4.306 | 0.000 | 0.6 |
| C3 | -4.820 | 0.000 | 0.7 | -2.171 | 0.030 | 0.3 | -0.012 | 0.991 | 0.0 | -5.112 | 0.000 | 0.8 | -5.555 | 0.000 | 0.8 |
| Cz | -4.318 | 0.000 | 0.7 | -0.619 | 0.536 | 0.1 | -1.202 | 0.229 | 0.2 | -5.065 | 0.000 | 0.8 | -5.777 | 0.000 | 0.9 |
| C4 | -4.505 | 0.000 | 0.7 | -2.124 | 0.034 | 0.3 | -1.074 | 0.283 | 0.2 | -4.575 | 0.000 | 0.7 | -4.528 | 0.000 | 0.7 |
| T4 | -4.621 | 0.000 | 0.7 | -4.131 | 0.000 | 0.6 | -0.595 | 0.552 | 0.1 | -4.131 | 0.000 | 0.6 | -4.271 | 0.000 | 0.6 |
| T5 | -5.180 | 0.000 | 0.8 | -2.964 | 0.003 | 0.4 | -1.424 | 0.155 | 0.2 | -4.936 | 0.000 | 0.7 | -5.532 | 0.000 | 0.8 |
| P3 | -4.411 | 0.000 | 0.7 | -1.821 | 0.069 | 0.3 | -2.859 | 0.004 | 0.4 | -4.376 | 0.000 | 0.7 | -5.777 | 0.000 | 0.9 |
| Pz | -4.353 | 0.000 | 0.7 | -2.276 | 0.023 | 0.3 | -2.719 | 0.007 | 0.4 | -3.921 | 0.000 | 0.6 | -5.777 | 0.000 | 0.9 |
| P4 | -4.376 | 0.000 | 0.7 | -1.552 | 0.121 | 0.2 | -2.567 | 0.010 | 0.4 | -3.828 | 0.000 | 0.6 | -5.777 | 0.000 | 0.9 |
| T6 | -3.571 | 0.000 | 0.5 | -2.451 | 0.014 | 0.4 | -2.101 | 0.036 | 0.3 | -4.843 | 0.000 | 0.7 | -5.520 | 0.000 | 0.8 |
| O1 | -4.516 | 0.000 | 0.7 | -4.143 | 0.000 | 0.6 | -1.774 | 0.076 | 0.3 | -4.715 | 0.000 | 0.7 | -5.777 | 0.000 | 0.9 |
| O2 | -3.910 | 0.000 | 0.6 | -3.816 | 0.000 | 0.6 | -1.669 | 0.095 | 0.3 | -4.598 | 0.000 | 0.7 | -5.777 | 0.000 | 0.9 |

Results of nonparametric Wilcoxon-tests per channel and frequency band. *r* = effect size.

| Table S2. Hypothesis 1b: Spectral EEG power comparisons between after haptic exploration (aHE) and before spontaneous facial self-touch (b-sFST) | | | | | | | | | | | | | | | |
| --- | --- | --- | --- | --- | --- | --- | --- | --- | --- | --- | --- | --- | --- | --- | --- |
| EEG electrodes | Delta | | | Theta | | | Alpha | | | Beta | | | Gamma | |  |
|  | z | *p* | *r* | z | *p* | *r* | z | *p* | *r* | z | *p* | *r* | z | *p* | *r* |
| Fp1 | -2.801 | 0.005 | 0.4 | -3.221 | 0.001 | 0.5 | -3.419 | 0.001 | 0.5 | -2.824 | 0.005 | 0.4 | -2.322 | 0.020 | 0.4 |
| Fp2 | -3.489 | 0.000 | 0.5 | -3.373 | 0.001 | 0.5 | -3.968 | 0.000 | 0.6 | -3.478 | 0.001 | 0.5 | -2.439 | 0.015 | 0.4 |
| F7 | -4.843 | 0.000 | 0.7 | -3.198 | 0.001 | 0.5 | -2.217 | 0.027 | 0.3 | -1.786 | 0.074 | 0.3 | -0.082 | 0.935 | 0.0 |
| F3 | -3.968 | 0.000 | 0.6 | -1.914 | 0.056 | 0.3 | -0.350 | 0.726 | 0.1 | -2.171 | 0.030 | 0.3 | -2.124 | 0.034 | 0.3 |
| Fz | -3.618 | 0.000 | 0.5 | -0.385 | 0.700 | 0.1 | -0.572 | 0.567 | 0.1 | -2.964 | 0.003 | 0.4 | -2.661 | 0.008 | 0.4 |
| F4 | -3.536 | 0.000 | 0.5 | -1.074 | 0.283 | 0.2 | -0.315 | 0.753 | 0.0 | -1.097 | 0.273 | 0.2 | -1.645 | 0.100 | 0.2 |
| F8 | -3.956 | 0.000 | 0.6 | -3.559 | 0.000 | 0.5 | -2.287 | 0.022 | 0.3 | -0.992 | 0.321 | 0.1 | -0.805 | 0.421 | 0.1 |
| T3 | -3.816 | 0.000 | 0.6 | -3.396 | 0.001 | 0.5 | -0.735 | 0.462 | 0.1 | -4.131 | 0.000 | 0.6 | -3.968 | 0.000 | 0.6 |
| C3 | -3.513 | 0.000 | 0.5 | -2.054 | 0.040 | 0.3 | -0.642 | 0.521 | 0.1 | -4.248 | 0.000 | 0.6 | -2.684 | 0.007 | 0.4 |
| Cz | -3.104 | 0.002 | 0.5 | -0.187 | 0.852 | 0.0 | -2.136 | 0.033 | 0.3 | -3.373 | 0.001 | 0.5 | -3.594 | 0.000 | 0.5 |
| C4 | -3.583 | 0.000 | 0.5 | -0.548 | 0.583 | 0.1 | -2.626 | 0.009 | 0.4 | -2.964 | 0.003 | 0.4 | -2.544 | 0.011 | 0.4 |
| T4 | -3.548 | 0.000 | 0.5 | -2.112 | 0.035 | 0.3 | -0.595 | 0.552 | 0.1 | -3.828 | 0.000 | 0.6 | -3.641 | 0.000 | 0.5 |
| T5 | -2.999 | 0.003 | 0.5 | -1.867 | 0.062 | 0.3 | -1.727 | 0.084 | 0.3 | -4.481 | 0.000 | 0.7 | -4.225 | 0.000 | 0.6 |
| P3 | -2.707 | 0.007 | 0.4 | -1.774 | 0.076 | 0.3 | -2.381 | 0.017 | 0.4 | -4.050 | 0.000 | 0.6 | -4.610 | 0.000 | 0.7 |
| Pz | -3.361 | 0.001 | 0.5 | -1.552 | 0.121 | 0.2 | -2.918 | 0.004 | 0.4 | -3.046 | 0.002 | 0.5 | -4.691 | 0.000 | 0.7 |
| P4 | -2.672 | 0.008 | 0.4 | -0.840 | 0.401 | 0.1 | -3.338 | 0.001 | 0.5 | -2.789 | 0.005 | 0.4 | -4.680 | 0.000 | 0.7 |
| T6 | -2.311 | 0.021 | 0.3 | -1.062 | 0.288 | 0.2 | -1.610 | 0.107 | 0.2 | -4.283 | 0.000 | 0.6 | -5.065 | 0.000 | 0.8 |
| O1 | -1.821 | 0.069 | 0.3 | -1.505 | 0.132 | 0.2 | -1.155 | 0.248 | 0.2 | -4.050 | 0.000 | 0.6 | -4.528 | 0.000 | 0.7 |
| O2 | -1.762 | 0.078 | 0.3 | -1.319 | 0.187 | 0.2 | -1.272 | 0.203 | 0.2 | -3.758 | 0.000 | 0.6 | -4.831 | 0.000 | 0.7 |

Results of nonparametric Wilcoxon-tests per channel and frequency band. *r* = effect size.

| Table S3. *Hypothesis 1c: Spectral EEG power comparisons between before spontaneous facial self-touch (b-sFST) and after sFST (a-sFST)* | | | | | | | | | | | | | | | |
| --- | --- | --- | --- | --- | --- | --- | --- | --- | --- | --- | --- | --- | --- | --- | --- |
| EEG electrodes | Delta | | | Theta | | | Alpha | | | Beta | | | Gamma | |  |
|  | z | *p* | *r* | z | *p* | *r* | z | *p* | *r* | z | *p* | *r* | z | *p* | *r* |
| Fp1 | -3.314 | .001 | 0.5 | -0.467 | .641 | 0.1 | -1.342 | .180 | 0.2 | -1.552 | .121 | 0.2 | -1.295 | .195 | 0.2 |
| Fp2 | -4.388 | .000 | 0.7 | -2.112 | .035 | 0.3 | -2.906 | .004 | 0.4 | -3.081 | .002 | 0.5 | -1.33 | .183 | 0.2 |
| F7 | -3.839 | .000 | 0.6 | -1.704 | .088 | 0.3 | -0.128 | .898 | 0.0 | -3.069 | .002 | 0.5 | -2.462 | .014 | 0.4 |
| F3 | -3.478 | .001 | 0.5 | -0.537 | .591 | 0.1 | -1.050 | .294 | 0.2 | -2.591 | .010 | 0.4 | -2.509 | .012 | 0.4 |
| Fz | -2.240 | .025 | 0.3 | -0.058 | .953 | 0.0 | -1.120 | .263 | 0.2 | -1.097 | .273 | 0.2 | -2.614 | .009 | 0.4 |
| F4 | -2.521 | .012 | 0.4 | -0.432 | .666 | 0.1 | -0.502 | .616 | 0.1 | -1.575 | .115 | 0.2 | -1.692 | .091 | 0.3 |
| F8 | -3.373 | .001 | 0.5 | -1.225 | .220 | 0.2 | -0.759 | .448 | 0.1 | -3.104 | .002 | 0.5 | -3.618 | .000 | 0.5 |
| T3 | -3.536 | .000 | 0.5 | -1.727 | .084 | 0.3 | -0.992 | .321 | 0.1 | -2.614 | .009 | 0.4 | -3.629 | .000 | 0.5 |
| C3 | -2.602 | .009 | 0.4 | -1.587 | .112 | 0.2 | -0.899 | .369 | 0.1 | -3.034 | .002 | 0.5 | -3.653 | .000 | 0.6 |
| Cz | -1.599 | .110 | 0.2 | -0.012 | .991 | 0.0 | -1.727 | .084 | 0.3 | -1.786 | .074 | 0.3 | -3.501 | .000 | 0.5 |
| C4 | -2.626 | .009 | 0.4 | -0.303 | .762 | 0.0 | -0.689 | .491 | 0.1 | -3.081 | .002 | 0.5 | -3.664 | .000 | 0.6 |
| T4 | -3.303 | .001 | 0.5 | -0.058 | .953 | 0.0 | -1.004 | .316 | 0.2 | -3.128 | .002 | 0.5 | -3.338 | .001 | 0.5 |
| T5 | -1.669 | .095 | 0.3 | -1.132 | .258 | 0.2 | -0.840 | .401 | 0.1 | -3.338 | .001 | 0.5 | -4.703 | .000 | 0.7 |
| P3 | -1.377 | .168 | 0.2 | -1.377 | .168 | 0.2 | -1.575 | .115 | 0.2 | -3.081 | .002 | 0.5 | -4.57 | .000 | 0.7 |
| Pz | -1.937 | .053 | 0.3 | -0.198 | .843 | 0.0 | -1.751 | .080 | 0.3 | -1.564 | .118 | 0.2 | -3.875 | .000 | 0.6 |
| P4 | -1.074 | .283 | 0.2 | -0.794 | .427 | 0.1 | -1.832 | .067 | 0.3 | -2.346 | .019 | 0.4 | -4.236 | .000 | 0.6 |
| T6 | -1.902 | .057 | 0.3 | -0.630 | .529 | 0.1 | -1.727 | .084 | 0.3 | -3.279 | .001 | 0.5 | -4.446 | .000 | 0.7 |
| O1 | -1.634 | .102 | 0.2 | -1.039 | .299 | 0.2 | -1.704 | .088 | 0.3 | -2.719 | .007 | 0.4 | -4.353 | .000 | 0.7 |
| O2 | -0.397 | .692 | 0.1 | -0.420 | .674 | 0.1 | -0.794 | .427 | 0.1 | -2.416 | .016 | 0.4 | -4.493 | .000 | 0.7 |

Results of non-parametric Wilcoxon-tests per channel and frequency band. *r* = effect size.

| Table S4. *Hypothesis 2a: Spectral EEG power comparisons between before spontaneous facial self-touch (b-sFST) and start of skin contact (c-start)* | | | | | | | | | | | | | | | |
| --- | --- | --- | --- | --- | --- | --- | --- | --- | --- | --- | --- | --- | --- | --- | --- |
| EEG electrodes | Delta | | | Theta | | | Alpha | | | Beta | | | Gamma | |  |
|  | z | *p* | *r* | z | *p* | *r* | z | *p* | *r* | z | *p* | *r* | z | *p* | *r* |
| Fp1 | -4.960 | .000 | 0.7 | -4.820 | .000 | 0.7 | -3.956 | .000 | 0.6 | -4.225 | .000 | 0.6 | -3.875 | .000 | 0.6 |
| Fp2 | -5.333 | .000 | 0.8 | -4.948 | .000 | 0.7 | -4.318 | .000 | 0.7 | -4.481 | .000 | 0.7 | -4.260 | .000 | 0.6 |
| F7 | -4.598 | .000 | 0.7 | -1.237 | .216 | 0.2 | -0.945 | .345 | 0.1 | -0.782 | .434 | 0.1 | -1.797 | .072 | 0.3 |
| F3 | -4.201 | .000 | 0.6 | -0.840 | .401 | 0.1 | -0.280 | .779 | 0.0 | -2.789 | .005 | 0.4 | -4.120 | .000 | 0.6 |
| Fz | -4.878 | .000 | 0.7 | -0.560 | .575 | 0.1 | -2.567 | .010 | 0.4 | -0.397 | .692 | 0.1 | -3.793 | .000 | 0.6 |
| F4 | -4.633 | .000 | 0.7 | -1.529 | .126 | 0.2 | -0.327 | .744 | 0.0 | -1.996 | .046 | 0.3 | -3.186 | .001 | 0.5 |
| F8 | -4.855 | .000 | 0.7 | -2.112 | .035 | 0.3 | -1.132 | .258 | 0.2 | -1.330 | .183 | 0.2 | -1.937 | .053 | 0.3 |
| T3 | -4.306 | .000 | 0.6 | -1.214 | .225 | 0.2 | -2.602 | .009 | 0.4 | -0.362 | .718 | 0.1 | -1.517 | .129 | 0.2 |
| C3 | -3.828 | .000 | 0.6 | -2.077 | .038 | 0.3 | -4.155 | .000 | 0.6 | -1.354 | .176 | 0.2 | -1.657 | .097 | 0.2 |
| Cz | -3.933 | .000 | 0.6 | -3.174 | .002 | 0.5 | -4.271 | .000 | 0.6 | -1.961 | .050 | 0.3 | -2.672 | .008 | 0.4 |
| C4 | -4.295 | .000 | 0.6 | -2.567 | .010 | 0.4 | -3.454 | .001 | 0.5 | -1.295 | .195 | 0.2 | -1.727 | .084 | 0.3 |
| T4 | -4.306 | .000 | 0.6 | -0.105 | .916 | 0.0 | -2.404 | .016 | 0.4 | -0.093 | .926 | 0.0 | -1.062 | .288 | 0.2 |
| T5 | -2.813 | .005 | 0.4 | -3.081 | .002 | 0.5 | -4.166 | .000 | 0.6 | -1.517 | .129 | 0.2 | -4.120 | .000 | 0.6 |
| P3 | -2.544 | .011 | 0.4 | -3.291 | .001 | 0.5 | -4.446 | .000 | 0.7 | -1.669 | .095 | 0.3 | -3.431 | .001 | 0.5 |
| Pz | -2.848 | .004 | 0.4 | -3.828 | .000 | 0.6 | -3.945 | .000 | 0.6 | -1.634 | .102 | 0.2 | -3.233 | .001 | 0.5 |
| P4 | -2.801 | .005 | 0.4 | -3.921 | .000 | 0.6 | -3.641 | .000 | 0.5 | -0.455 | .649 | 0.1 | -3.338 | .001 | 0.5 |
| T6 | -2.416 | .016 | 0.4 | -3.046 | .002 | 0.5 | -1.984 | .047 | 0.3 | -1.751 | .080 | 0.3 | -4.318 | .000 | 0.7 |
| O1 | -1.424 | .155 | 0.2 | -3.431 | .001 | 0.5 | -3.349 | .001 | 0.5 | -0.677 | .498 | 0.1 | -4.551 | .000 | 0.7 |
| O2 | -1.587 | .112 | 0.2 | -3.163 | .002 | 0.5 | -2.299 | .022 | 0.3 | -1.179 | .239 | 0.2 | -4.785 | .000 | 0.7 |

Results of non-parametric Wilcoxon-tests per channel and frequency band. *r* = effect size.

| Table S5. Hypothesis 2b: Spectral EEG power comparisons between start of skin contact (c-start) and end of skin contact (c-end) of a spontaneous facial self-touch | | | | | | | | | | | | | | | |
| --- | --- | --- | --- | --- | --- | --- | --- | --- | --- | --- | --- | --- | --- | --- | --- |
| EEG electrodes | Delta | | | Theta | | | Alpha | | | Beta | | | Gamma | |  |
|  | z | *p* | *r* | z | *p* | *r* | z | *p* | *r* | z | *p* | *r* | z | *p* | *r* |
| Fp1 | -0.957 | .339 | 0.1 | -1.867 | .062 | 0.3 | -1.074 | .283 | 0.2 | -1.540 | .123 | 0.2 | -0.292 | .770 | 0.0 |
| Fp2 | -2.311 | .021 | 0.3 | -2.194 | .028 | 0.3 | -2.066 | .039 | 0.3 | -1.074 | .283 | 0.2 | -0.222 | .825 | 0.0 |
| F7 | -0.467 | .641 | 0.1 | -0.957 | .339 | 0.1 | -0.327 | .744 | 0.0 | -0.058 | .953 | 0.0 | -0.934 | .351 | 0.1 |
| F3 | -0.840 | .401 | 0.1 | -0.794 | .427 | 0.1 | -1.610 | .107 | 0.2 | -0.035 | .972 | 0.0 | -0.058 | .953 | 0.0 |
| Fz | -1.739 | .082 | 0.3 | -0.595 | .552 | 0.1 | -0.198 | .843 | 0.0 | -0.397 | .692 | 0.1 | -0.362 | .718 | 0.1 |
| F4 | -1.202 | .229 | 0.2 | -1.984 | .047 | 0.3 | -0.525 | .599 | 0.1 | -0.513 | .608 | 0.1 | -0.724 | .469 | 0.1 |
| F8 | -0.677 | .498 | 0.1 | -1.190 | .234 | 0.2 | -0.630 | .529 | 0.1 | -0.303 | .762 | 0.0 | -1.284 | .199 | 0.2 |
| T3 | -0.047 | .963 | 0.0 | -0.432 | .666 | 0.1 | -1.657 | .097 | 0.2 | -0.187 | .852 | 0.0 | -0.490 | .624 | 0.1 |
| C3 | -1.027 | .304 | 0.2 | -1.295 | .195 | 0.2 | -0.047 | .963 | 0.0 | -1.762 | .078 | 0.3 | -0.023 | .981 | 0.0 |
| Cz | -1.412 | .158 | 0.2 | -1.144 | .253 | 0.2 | -0.443 | .657 | 0.1 | -0.922 | .357 | 0.1 | -0.198 | .843 | 0.0 |
| C4 | -1.389 | .165 | 0.2 | -1.325 | .185 | 0.2 | -0.268 | .788 | 0.0 | -0.724 | .469 | 0.1 | -0.152 | .879 | 0.0 |
| T4 | -0.385 | .700 | 0.1 | -0.350 | .726 | 0.1 | -0.945 | .345 | 0.1 | -0.887 | .375 | 0.1 | -1.027 | .304 | 0.2 |
| T5 | -0.397 | .692 | 0.1 | -0.292 | .770 | 0.0 | -1.447 | .148 | 0.2 | -1.085 | .278 | 0.2 | -1.354 | .176 | 0.2 |
| P3 | -1.377 | .168 | 0.2 | -0.467 | .641 | 0.1 | -0.128 | .898 | 0.0 | -1.482 | .138 | 0.2 | -0.852 | .394 | 0.1 |
| Pz | -1.330 | .183 | 0.2 | -0.922 | .357 | 0.1 | -0.548 | .583 | 0.1 | -0.128 | .898 | 0.0 | -0.315 | .753 | 0.0 |
| P4 | -1.809 | .070 | 0.3 | -0.70 | .484 | 0.1 | -1.085 | .278 | 0.2 | -0.548 | .583 | 0.1 | -0.478 | .632 | 0.1 |
| T6 | -0.082 | .935 | 0.0 | -0.222 | .825 | 0.0 | -0.969 | .333 | 0.1 | -0.128 | .898 | 0.0 | -0.654 | .513 | 0.1 |
| O1 | -0.420 | .674 | 0.1 | -0.338 | .735 | 0.1 | -1.412 | .158 | 0.2 | -0.467 | .641 | 0.1 | -0.210 | .834 | 0.0 |
| O2 | -0.070 | .944 | 0.0 | -0.152 | .879 | 0.0 | -0.012 | .991 | 0.0 | -1.144 | .253 | 0.2 | -0.724 | .469 | 0.1 |

Results of nonparametric Wilcoxon-tests per channel and frequency band. *r* = effect size.

| Table S6. *Hypothesis 2c: Spectral EEG power comparisons between end of skin contact (c-end) and after spontaneous facial self-touch (a-sFST)* | | | | | | | | | | | | | | | |
| --- | --- | --- | --- | --- | --- | --- | --- | --- | --- | --- | --- | --- | --- | --- | --- |
| EEG electrodes | Delta | | | Theta | | | Alpha | | | Beta | | | Gamma | |  |
|  | z | *p* | *r* | z | *p* | *r* | z | *p* | *r* | z | *p* | *r* | z | *p* | *r* |
| Fp1 | -4.621 | .000 | 0.7 | -3.956 | .000 | 0.6 | -3.431 | .001 | 0.5 | -4.003 | .000 | 0.6 | -4.131 | .000 | 0.6 |
| Fp2 | -4.061 | .000 | 0.6 | -3.594 | .000 | 0.5 | -3.058 | .002 | 0.5 | -3.466 | .001 | 0.5 | -4.225 | .000 | 0.6 |
| F7 | -3.268 | .001 | 0.5 | -0.397 | .692 | 0.1 | -0.864 | .388 | 0.1 | -1.867 | .062 | 0.3 | -0.152 | .879 | 0.0 |
| F3 | -3.139 | .002 | 0.5 | -1.132 | .258 | 0.2 | -0.805 | .421 | 0.1 | -1.762 | .078 | 0.3 | -3.513 | .000 | 0.5 |
| Fz | -2.404 | .016 | 0.4 | -2.427 | .015 | 0.4 | -2.451 | .014 | 0.4 | -0.093 | .926 | 0.0 | -2.509 | .012 | 0.4 |
| F4 | -3.174 | .002 | 0.5 | -0.373 | .709 | 0.1 | -1.459 | .145 | 0.2 | -1.575 | .115 | 0.2 | -3.174 | .002 | 0.5 |
| F8 | -2.929 | .003 | 0.4 | -0.233 | .815 | 0.0 | -0.642 | .521 | 0.1 | -0.584 | .560 | 0.1 | -0.222 | .825 | 0.0 |
| T3 | -1.704 | .088 | 0.3 | -2.801 | .005 | 0.4 | -3.209 | .001 | 0.5 | -1.214 | .225 | 0.2 | -0.513 | .608 | 0.1 |
| C3 | -1.716 | .086 | 0.3 | -3.688 | .000 | 0.6 | -4.131 | .000 | 0.6 | -2.229 | .026 | 0.3 | -0.012 | .991 | 0.0 |
| Cz | -1.494 | .135 | 0.2 | -4.155 | .000 | 0.6 | -4.108 | .000 | 0.6 | -2.567 | .010 | 0.4 | -0.268 | .788 | 0.0 |
| C4 | -1.435 | .151 | 0.2 | -3.875 | .000 | 0.6 | -4.236 | .000 | 0.6 | -2.497 | .013 | 0.4 | -0.385 | .700 | 0.1 |
| T4 | -0.770 | .441 | 0.1 | -1.109 | .268 | 0.2 | -2.509 | .012 | 0.4 | -1.365 | .172 | 0.2 | -1.155 | .248 | 0.2 |
| T5 | -1.821 | .069 | 0.3 | -3.828 | .000 | 0.6 | -3.688 | .000 | 0.6 | -0.584 | .560 | 0.1 | -1.295 | .195 | 0.2 |
| P3 | -0.280 | .779 | 0.0 | -4.691 | .000 | 0.7 | -4.073 | .000 | 0.6 | -1.867 | .062 | 0.3 | -0.770 | .441 | 0.1 |
| Pz | -0.362 | .718 | 0.1 | -4.890 | .000 | 0.7 | -3.921 | .000 | 0.6 | -2.871 | .004 | 0.4 | -0.175 | .861 | 0.0 |
| P4 | -0.630 | .529 | 0.1 | -3.431 | .001 | 0.5 | -3.466 | .001 | 0.5 | -1.832 | .067 | 0.3 | -0.268 | .788 | 0.0 |
| T6 | -0.175 | .861 | 0.0 | -2.661 | .008 | 0.4 | -2.287 | .022 | 0.3 | -0.012 | .991 | 0.0 | -0.735 | .462 | 0.1 |
| O1 | -0.467 | .641 | 0.1 | -4.761 | .000 | 0.7 | -2.241 | .025 | 0.3 | -0.408 | .683 | 0.1 | -0.700 | .484 | 0.1 |
| O2 | -0.957 | .339 | 0.1 | -3.711 | .000 | 0.6 | -1.949 | .051 | 0.3 | -0.443 | .657 | 0.1 | -0.397 | .692 | 0.1 |

Results of non-parametric Wilcoxon-tests per channel and frequency band. *r* = effect size.
